# Maternal obesity alters the placental transcriptome in a fetal sex-dependent manner

**DOI:** 10.3389/fcell.2023.1178533

**Published:** 2023-06-15

**Authors:** Amy Kelly, Jeannie Chan, Theresa L. Powell, Laura A. Cox, Thomas Jansson, Fredrick J. Rosario

**Affiliations:** ^1^ Department of Surgery, University of Arizona College of Medicine, Tucson, AZ, United States; ^2^ Division of Reproductive Sciences, Department of Obstetrics and Gynecology, University of Colorado Anschutz Medical Campus, Aurora, CO, United States; ^3^ Center for Precision Medicine, Department of Internal Medicine, Section of Molecular Medicine, Wake Forest School of Medicine, Winston-Salem, NC, United States; ^4^ Section of Neonatology, Department of Pediatrics, University of Colorado Anschutz Medical Campus, Aurora, CO, United States

**Keywords:** trophoblast, maternal–fetal exchange, gene expression, mitochondria, fetal growth

## Abstract

Infants born to obese mothers have an increased risk of developing obesity and metabolic diseases in childhood and adulthood. Although the molecular mechanisms linking maternal obesity during pregnancy to the development of metabolic diseases in offspring are poorly understood, evidence suggests that changes in the placental function may play a role. Using a mouse model of diet-induced obesity with fetal overgrowth, we performed RNA-seq analysis at embryonic day 18.5 to identify genes differentially expressed in the placentas of obese and normal-weight dams (controls). In male placentas, 511 genes were upregulated and 791 genes were downregulated in response to maternal obesity. In female placentas, 722 genes were downregulated and 474 genes were upregulated in response to maternal obesity. The top canonical pathway downregulated in maternal obesity in male placentas was oxidative phosphorylation. In contrast, sirtuin signaling, NF-kB signaling, phosphatidylinositol, and fatty acid degradation were upregulated. In female placentas, the top canonical pathways downregulated in maternal obesity were triacylglycerol biosynthesis, glycerophospholipid metabolism, and endocytosis. In contrast, bone morphogenetic protein, TNF, and MAPK signaling were upregulated in the female placentas of the obese group. In agreement with RNA-seq data, the expression of proteins associated with oxidative phosphorylation was downregulated in male but not female placentas of obese mice. Similarly, sex-specific changes in the protein expression of mitochondrial complexes were found in placentas collected from obese women delivering large-for-gestational-age (LGA) babies. In conclusion, maternal obesity with fetal overgrowth differentially regulates the placental transcriptome in male and female placentas, including genes involved in oxidative phosphorylation.

## Introduction

The worldwide obesity epidemic constitutes a significant public health challenge among pregnant women (Kim et al., 2007). The prevalence of obesity in US women of reproductive age (18–49) increased from 7.4% in 1976 to 27.5% in 2014 (Ogden et al., 2012; Fisher et al., 2013; Ogden et al., 2013; Ogden et al., 2014; National Center for Health Statistics, 2015). Maternal obesity during pregnancy is associated with various pregnancy complications, including gestational diabetes mellitus (GDM) and preeclampsia (Marchi et al., 2015). In addition, obese women have an increased risk of delivering a large-for-gestational-age (LGA) infant (Yu et al., 2013; Leon-Garcia et al., 2016). Infants of obese mothers are more likely to have poor neonatal outcomes and a greater risk of developing obesity, cancer, insulin resistance, hypertension, and dyslipidemia later in life (Evagelidou et al., 2006; Wang et al., 2007; Alfaradhi and Ozanne, 2011; Bouret et al., 2015). Maternal obesity in women is associated with premature death in adult offspring (Reynolds et al., 2013). Furthermore, animal experiments have shown that diet-induced maternal obesity causes permanent dysregulation of food intake (Kirk et al., 2009), hyperphagia (Samuelsson et al., 2008), adiposity (Samuelsson et al., 2008), non-alcoholic fatty liver (Oben et al., 2010), myocardial dysfunction (Samuelsson et al., 2013), and hypertension (Samuelsson et al., 2010; Taylor et al., 2014) in adult offspring. The molecular mechanisms linking maternal obesity during pregnancy to adverse long-term outcomes in children are poorly understood. However, emerging evidence suggests that changes in the placental structure and function may be involved (Roberts et al., 2011; Wallace et al., 2012; Jansson et al., 2013; Burton et al., 2016; Mitsuya et al., 2017).

The placenta integrates an array of signals from both the mother and fetus to maintain fetal homeostasis (Jansson and Powell, 2013; Diaz et al., 2014). It performs numerous functions critical for normal fetal growth and development, including mediating nutrient and waste transfer, secreting hormones, serving as an immunological barrier, and performing xenobiotic detoxification (Burton et al., 2016). Placental nutrient transport capacity has been reported to be increased in obese women delivering LGA infants and in animal models of diet-induced maternal obesity (Jansson et al., 2013; Aye et al., 2015; Rosario et al., 2015). We developed a mouse model of maternal obesity during pregnancy induced by a high-fat/high-sugar diet, showing extensive similarities to the human condition, including elevated levels of maternal leptin, glucose intolerance, activation of placental insulin and mTOR signaling, increased placental nutrient transport, and fetal overgrowth (Aye et al., 2015; Rosario et al., 2015). Significantly, offspring of obese mice developed metabolic (Paulsen et al., 2019; Dumolt et al., 2022) and cardiovascular diseases in adulthood (Vaughan et al., 2020; Vaughan et al., 2022). Nam and co-workers reported an increased placental expression of nutrient transporters, activation of placental mTOR signaling, and fetal overgrowth in a mouse model of maternal high-fat diet (Nam et al., 2017). These observations are consistent with the possibility that enhanced the placental transfer of nutrients, contributing to fetal overgrowth in maternal obesity.

Experimental animal and human epidemiological studies have demonstrated a sex difference in response to an adverse intrauterine milieu and the subsequent risk of developing diseases later in life (Zhu et al., 2018; Alves et al., 2020). Moreover, gene expression is distinct in male and female placentas (Clifton, 2010; Osei-Kumah et al., 2011; Cox et al., 2013; Barke et al., 2019). Male fetuses grow faster than female fetuses *in utero*, which is believed to place male fetuses at a greater risk in an adverse environment, possibly explaining the increased incidence of adverse perinatal outcomes in male fetuses. Furthermore, emerging evidence suggests that female fetuses adapt better than male fetuses to an adverse intrauterine environment, which could contribute to better perinatal and long-term outcomes in female fetuses, as demonstrated in some studies. Using a mouse model of maternal obesity with fetal overgrowth, we recently demonstrated that male offspring develop obesity, glucose intolerance, and insulin resistance at 3 months of age, whereas the metabolic phenotype in female offspring is much less severe at this age (Paulsen et al., 2019). However, the molecular mechanisms underlying sex-dependent *in utero* growth strategies are largely unknown.

Maternal obesity affects mitochondrial biogenesis, oxidative stress, and antioxidant activity in the placenta in a sexually dimorphic manner (Mele et al., 2014; Evans and Myatt, 2017). Despite the proposed important role of the placenta in mediating the adverse effects of maternal obesity on the long-term health of LGA infants, reports of the impact of maternal obesity on the placental transcriptome in pregnancies with fetal overgrowth are limited (Cox et al., 2019). To address this gap in knowledge, we characterized the placental transcriptome in our mouse model of diet-induced maternal obesity during pregnancy, which is associated with moderate fetal overgrowth and sexual dimorphism in long-term metabolic outcomes.

## Materials and methods

### Animals and diets

Institutional Animal Care and Use Committee review boards at the University of Texas Health Science Center in San Antonio approved all experimental protocols. Female C57BL/6 J mice (Jackson Laboratory, Bar Harbor, ME, United States of America), which were proven to be breeders with one previous litter, about 12 weeks of age were housed under controlled conditions (25°C, 12-h light/dark cycle). At 13 weeks of age, animals were randomly assigned to a control (D12489B, 10.6 kcal% fat) or high-fat pellet diet (Western Diet D12079B, 41 kcal% fat) with *ad libitum* access to a 20% sucrose solution (high-fat/high-sugar, HF/HS; obese). Sucrose solution was supplemented with vitamins (10 g/4,000 kcal Vitamin Mix V10001) and minerals (35 g/4,000 kcal Mineral Mix S10001). Diets were purchased from Research Diets (New Brunswick, NJ, United States). All animals had free access to water. When female mice on the HF/HS diet had a 25% increase in body weight, obese female mice and age-matched female mice on the control diet were mated with male C57BL/6 J mice on the control diet. Mating was confirmed via the presence of a vaginal plug (embryonic day (E) 0.5), and dams were continued on the respective diets throughout gestation. Body weight increased in pregnant mice at different gestational intervals (Supplementary Figure S1). At E18.5, dams were euthanized for the collection of placentas and fetuses. The weights of fetuses and placentas were recorded. Placentas for molecular analysis were immediately snap-frozen in liquid N_2_ and stored at −80°C.

The sex of fetuses was determined by PCR amplification (Hacker et al., 1995) of a fragment of the Y-linked *Zfy-1* gene using genomic DNA isolated from fetal tails by the HotSHOT method (Truett et al., 2000). The primers 5′-TGC​TGT​TAC​ATG​TTG​ACC​TGG-3′ and 5′-TGC​TCT​CCT​GTC​TCT​CCA​AGA-3′ amplified a 451-bp PCR product to indicate the presence of the Y chromosome. Amplicons were loaded on 2% agarose gels and electrophoresed together with a 100-bp DNA ladder. Under UV illumination, bands were visualized using an ethidium bromide gel stain, and the genotypic sex was identified according to the presence or absence of the *Zfy-1* gene.

### RNA extraction

After homogenizing placental tissues (∼10 mg) in the TRI reagent, total RNA was purified using the Direct-zol RNA MiniPrep Kits (Zymo Research, Irvine, CA), according to the instructions provided by the manufacturer. Purified RNA was resuspended in 50 µL DNase/RNase-free water. RNA quality was evaluated using an Agilent 2100 Bioanalyzer (Agilent Technologies, Inc., Santa Clara, CA). RNA concentrations were quantified using Qubit RNA HS assay kits and a Qubit 2.0 Fluorometer (Thermo Fisher Scientific, Wilmington, DE) and stored at −80°C until further analysis.

### RNA sequencing

One microgram of total RNA was depleted of ribosomal RNA, and PolyA tails of coding RNAs were captured by treatment with oligo-dT beads. Complementary DNA (cDNA) libraries were generated according to the TruSeq Stranded Total RNA sample preparation guide (Illumina, San Diego, CA, United States) and quantified using the KAPA Library Quantification Kit. Multiplex paired-end sequencing was performed using the Illumina HiSeq 2500 instrument (Illumina). Sequence reads were demultiplexed using the CASAVA pipeline (Illumina), and bases with Phred<30 were trimmed from both ends. Paired-end reads were aligned using STAR (PMID: 23104886) against the *Mus musculus* genome (GRCm38). Aligned reads were normalized by the TMM method (PMID: 20196867). After normalization, the gene-specific analysis tool in Partek Flow (Partek Inc., Chesterfield, MO) was used to identify differentially expressed genes (DEGs) by comparing expression in obese vs. control placentas.

### KEGG pathway enrichment analysis

Kyoto Encyclopedia of Genes and Genomes (KEGG) pathway enrichment analysis was performed separately for DEGs from male and female placentas using DAVID and GSEA tools (add websites of the online tools). They were entered separately; the cutoff criteria to identify enriched pathways were *p* < 0.05 and FDR <0.01 for DAVID, and nominal *p* < 0.05 and FDR<0.25 for GSEA. A Venn diagram in InteractiVenn (http://www.interactivenn.net/) was used to show the common enriched KEGG pathways between DAVID and GSEA analyses (Heberle et al., 2015).

### Gene ontology and functional analyses

DEGs of both male and female placentas were further characterized by gene ontology (GO) and pathway enrichment analyses. Over-represented GO cellular components, biological processes, molecular functions, and KEGG pathways were identified by using WebGestalt, with *p*-value <0.05 as the criterion for statistical significance after Benjamini–Hochberg correction for multiple testing (Supek et al., 2011). The REVIGO tool was used to filter out redundant GO terms, and enrichment analysis results were visualized using a treemap (Cox et al., 2019). Furthermore, functional analysis was carried out using the ingenuity pathway analysis (IPA, QIAGEN Inc.)

### Human placental samples

This study was approved by the Colorado Multiple Institutional Review Board. Women with uncomplicated singleton term (37–40 weeks gestation) pregnancies were recruited, with a written consent prior to delivery. The gestational age was estimated from the date of the last menstrual period and confirmed by ultrasound dating. A total of 36 women with varying BMI (range, 18.4–47.1 kg/m^2^) were studied. BMI was calculated based on height and weight measurements from pre-pregnancy medical records. The clinical characteristics of the participants in the study are shown in Table 1. Exclusion criteria for both AGA and obese/LGA groups were smoking, concurrent diseases, such as diabetes or hypertension, development of pregnancy complications, including gestational diabetes, pregnancy-induced hypertension, and pre-eclampsia, and the delivery of small-for-gestational-age (<10th centile) infants, according to published growth curves. Placental tissues were collected and processed within 30 min of delivery. In brief, the decidua basalis and chorionic plate were removed, and villous tissue was dissected and rinsed in cold physiological saline. The villous tissue was transferred to cold buffer D (250 mM sucrose, 10 mM HEPES, pH 7.4) containing 1:100 dilution of protease and phosphatase inhibitors (Sigma-Aldrich, St. Louis, MO) and homogenized on ice with Polytron (Kinematica, Luzern, Switzerland). The placental homogenates were frozen in liquid nitrogen and stored at −80°C until further processing. Mitochondrial electron transport complexes were determined using Western blots, as described in the following paragraph.

**TABLE 1:** Selected clinical data.

	AGA (male fetus)	Obese (male fetus)	AGA (female fetus)	Obese (female fetus)
Maternal age (years)	26.4 ± 2.2 (18–35)	30.7 ± 1.7 (21–40)	29.0 ± 2.3 (21–40)	33.3 ± 1.23 (21–40)
BMI (kg/m^2^) ^a^	22.5 ± 0.7^a^ (18.7–24.3)	34.2 ± 1.7^b^ (28.2–47.1)	23.1 ± 0.5^a^ (21.6–24.8)	38.0 ± 1.7^b^ (26.9–43.6)
Gestational age (weeks)	39.2 ± 0.28 (37.5–41.6)	38.9 ± 0.44 (37.1–41)	39.0 ± 0.25 (37–40)	38.9 ± 0.27 (37.6–39.4)
Birth weight (kg)	2.9 ± 0.03^a^ (2.7–2.9)	4.3 ± 0.19^b^ (3.6–5.9)	2.8 ± 0.06^a^ (2.7–3.0)	4.1 ± 0.09^b^ (3.8–4.5)
Birth weight percentile^b^	19.8 ± 4.1^a^ (6–41)	90.2 ± 2.8^b^ (73–100)	23.8 ± 5.0^a^ (11–46)	92.8 ± 2.5^b^ (77–99)
Placental weight (g)	559 ± 37^a^ (435–678)	851 ± 47^b^ (648–1.15)	518 ± 29^a^ (437–625)	854 ± 54^b^ (608–1.20)
Fetal/placental ratio	5.25 ± 0.27 (4.36–6.24)	5.12 ± 0.20 (4.35–6.70)	5.64 ± 0.23 (4.75–6.22)	5.00 ± 0.28 (3.74–6.96)

Data are presented as means ± S.E.M; range.

^a^ Abbreviations: data from n = AGA (male), 7; obese (male), 11; AGA (female), 6; obese (female), 10.

^b^ By corresponding gestational age; means without a common letter differ significantly (*p* < 0.05) by one-way ANOVA, with the Tukey–Kramer multiple comparison *post hoc* test.

### Western blotting

The total protein concentration in placental homogenates was determined using the Bradford assay (Bio-Rad). The placental homogenate (10 μg) was separated on 4%–20% precast linear gradient gels (Invitrogen). Membranes were incubated overnight at 4°C with primary antibody diluted in 1% non-fat milk (wt/vol) in TBST and detected using an appropriate peroxidase-conjugated secondary antibody. Products were visualized by ECL chemiluminescence (Millipore). Band intensities were measured using the G-box system (Syngene). Anti-β actin was purchased from Sigma-Aldrich, St. Louis, Mo. Target band densities were normalized using beta-actin to account for any variation in loading and transfer. Additional protein expression analysis was performed using automated capillary-based immunoassay (ProteinSimple, San Jose, CA, catalog #SM-W004-1, #PS-ST01, and #PN-009–050), as described previously (Castillo-Castrejon et al., 2021). Briefly, Jess plates were run according to the manufacturer’s instructions, with minor modification (200 V, 55 min separation time) with 0.1 mg/mL total protein concentration.

### Data presentation and statistics

Results from experimental analyses in mice are presented as mean ± SEM. The number of experiments (n) represents the number of litters studied. For each litter, male or female placentas were pooled. Results from human placentas are presented as mean ± SEM. The number of experiments (n) represents the number of individual placentas studied. Variables were analyzed as continuous across the range of BMI and birth weights, and linear relationships between variables were determined using Pearson’s correlation coefficients. A *p* < 0.05 value was considered significant. Statistical significance of differences between control and obese groups was assessed using Student’s *t*-test. A *p*-value < 0.05 was considered significant. One-way analysis of variance (ANOVA) and Tukey–Kramer multiple comparisons *post hoc* tests were used to compare datasets with more than two groups. *p* < 0.05 was considered significant.

## Results

### Fetal and placental weights in the mouse model of maternal obesity

**FIGURE 1:**
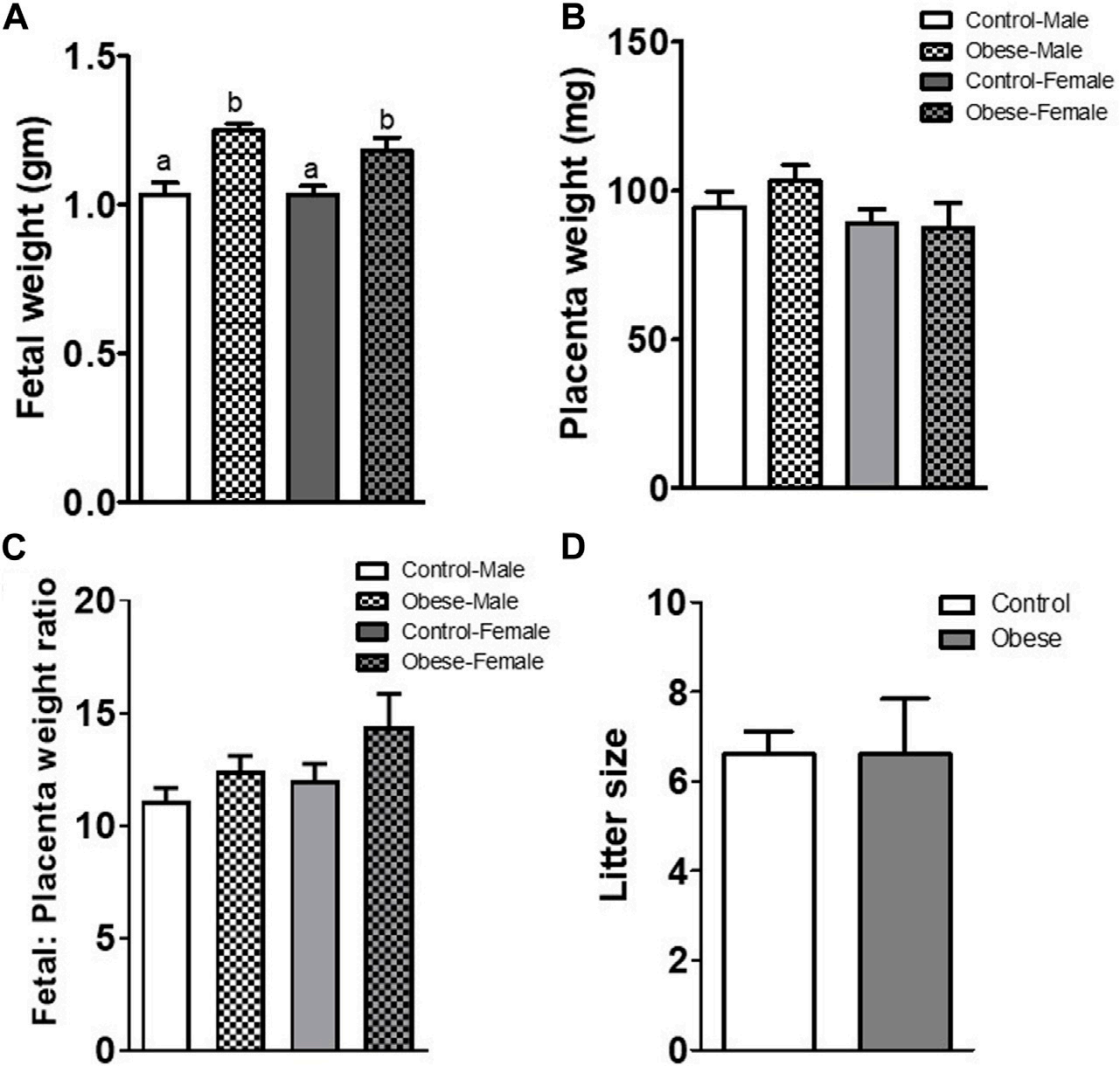
Fetal **(A)** and placental **(B)** weights, fetal/placental weight ratio **(C)**, and litter size **(D)** determined at E18.5 in mice from the control and obese groups. Values are means ± SEM, n = 5 in each group, *p < 0.05 vs control; unpaired Student’s *t*-test. Means without a common letter are statistically different by performing one-way ANOVA with the Tukey–Kramer multiple comparison *post hoc* test (*p* < 0.05).

Both male and female fetal weights were increased (*p* < 0.001; n = 5 litters in each group) at E18.5 in the obese group as compared to control (Figure 1A). However, placental weights and fetal/placental weight ratios were not significantly different between groups (Figures 1B, C). The increase in fetal weight was not due to a difference in the litter size (Figure 1D), which was similar in control (6.6 ± 0.51, n = 5) and obese groups (6.5 ± 1.2, n = 5).

### Analysis of differentially expressed genes

**FIGURE 2:**
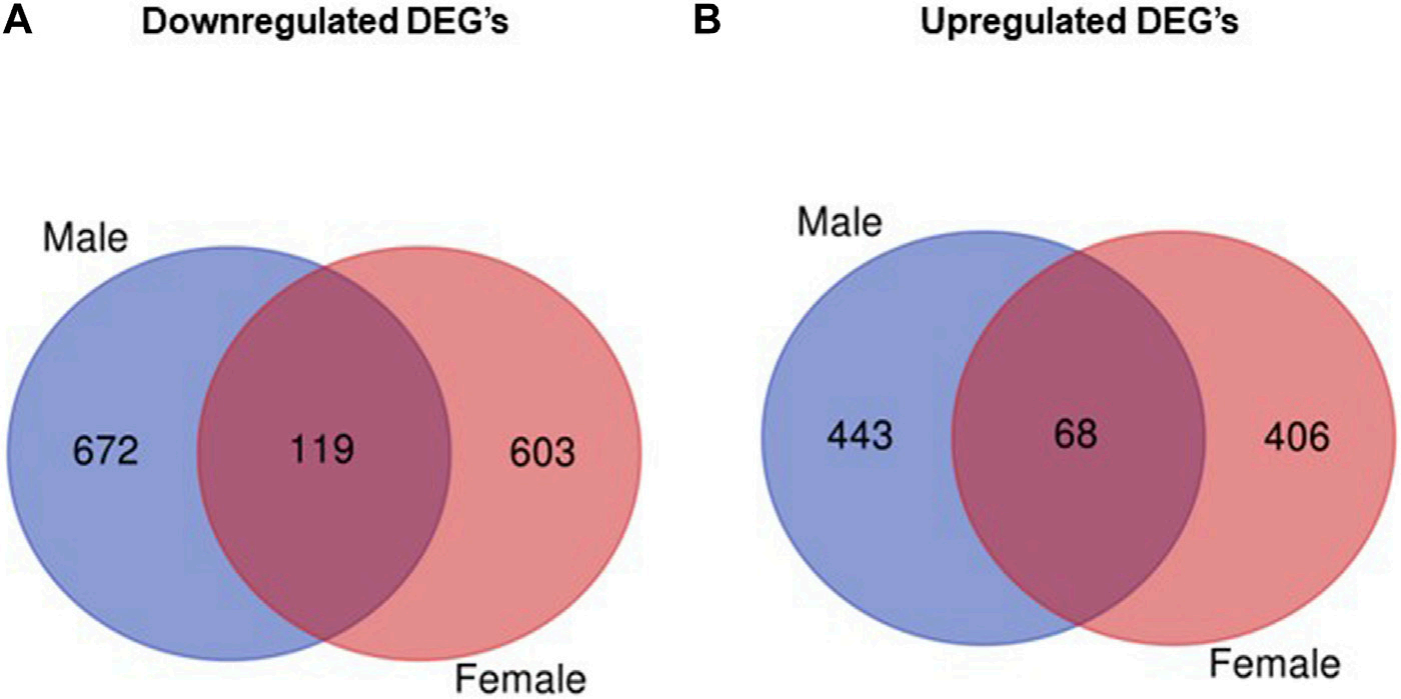
Venn diagram illustrating differentially expressed genes that overlap between males and females. **(A)** Number of downregulated genes (obese vs. control) in male and female placentas. **(B)** Number of upregulated genes (obese vs. control) in male and female placentas.

The analysis of RNA-seq data comparing gene expression in obese vs. control placentas without taking the fetal sex into account showed that of 25,099 transcripts (Supplementary Table S1), 1154 were significantly upregulated and 1133 were significantly downregulated (7.6% DEG, *p* < 0.05; Supplementary Table S1) in the obese group. When data were analyzed separately for placentas of male and female fetuses, RNA-seq identified 25,099 transcripts in male placentas with 511 genes significantly upregulated and 791 genes significantly downregulated when comparing the obese group with control (Supplementary Table S2). In female placentas, 25,099 genes were expressed, with 474 genes significantly upregulated and 722 genes significantly downregulated in the obese group as compared with the control group (Supplementary Table S3). Comparing placental DEGs in the mouse maternal obesity model, we observed that 119 DEGs were downregulated in both male and female fetuses (Figure 2; Supplementary Table S4) and 68 DEGs were upregulated in both sexes (Figure 2; Supplementary Table S5).

The top five downregulated genes in male placentas of obese dams were Nudix hydrolase 13 (*Nudt13*, 1080-fold); glutathione S-transferase A3 (*Gsta3*, 233-fold); thromboxane A2 receptor (*Tbxa2r*, 201-fold); roundabout homolog 2 (*Robo2*, 54-fold); and zinc finger and BTB domain-containing protein 7B (*Zbtb7b*, 28-fold). In female placentas of obese dams, the top five downregulated genes were aquaporin-5 (*Aqp5*, 8163-fold); ankyrin repeat family A protein 2 (*Ankra2*, 2896-fold); threonine synthase-like 1 (*Thnsl1*, 2122-fold); zyxin (*Zyx*, 1204-fold); and T-lymphocyte surface antigen Ly-9 (*Ly9*, 913-fold).

The top five upregulated genes in male placentas of obese dams as compared to the control group were myosin light chain 1/3, skeletal muscle isoform (*Myl1*, 12,571-fold); apolipoprotein C-III (*Apoc3*, 6177-fold); kerman protein 2 (*Kremen2*, 5053-fold); disabled homolog 2-interacting protein (*Dab2ip*, 2112-fold); and protein AF-9 (*Mllt3*, 180-fold). In female placentas of obese dams, the top five upregulated genes as compared to the control group were myosin light chain 1/3, skeletal muscle isoform (*Myl1*, 13,482-fold); myosin regulatory light chain 2, skeletal muscle isoform (*Mylpf*, 2381-fold) (*Zmynd15*, 467-fold); RWD domain-containing protein 2A (*Rwdd2a*, 250-fold); and tripartite motif protein 15 isoform (*Trim15*, 223-fold).

### GO enrichment analysis for upregulated genes in male placentas of obese dams

**FIGURE 3:**
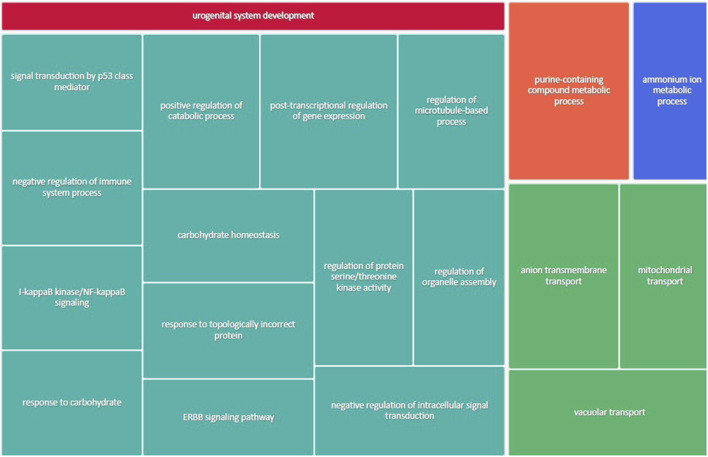
Treemap of GO biological process categories upregulated in male placentas of obese dams. REVIGO was used to remove redundant GO terms and join the cluster representatives (single rectangles) into superclusters (represented by different colors). Each rectangle’s size reflects the GO term’s false discovery rate (FDR) value (larger for lower FDR), considering a FDR <0.05 as the criterion for statistical significance after Benjamini–Hochberg correction for multiple testing.

**FIGURE 4:**
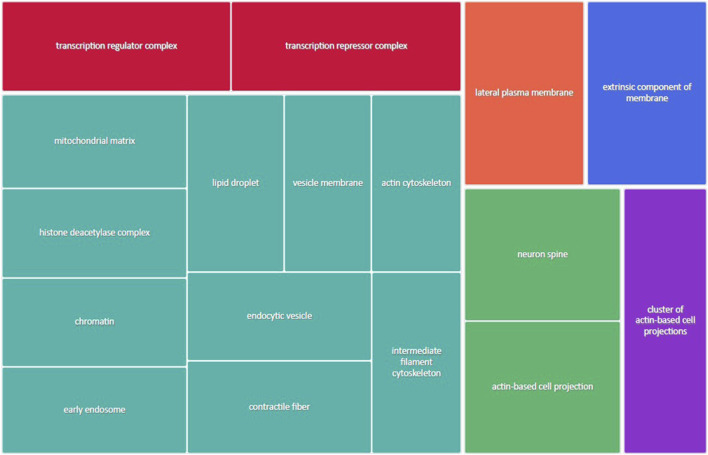
Treemap of GO cellular component categories upregulated in male placentas of obese dams. REVIGO was used to remove redundant GO terms and join the cluster representatives (single rectangles) into superclusters (represented by different colors). Each rectangle’s size reflects the GO term’s false discovery rate (FDR) value (larger for lower FDR), considering a FDR <0.05 as the criterion for statistical significance after Benjamini–Hochberg correction for multiple testing.

**FIGURE 5:**
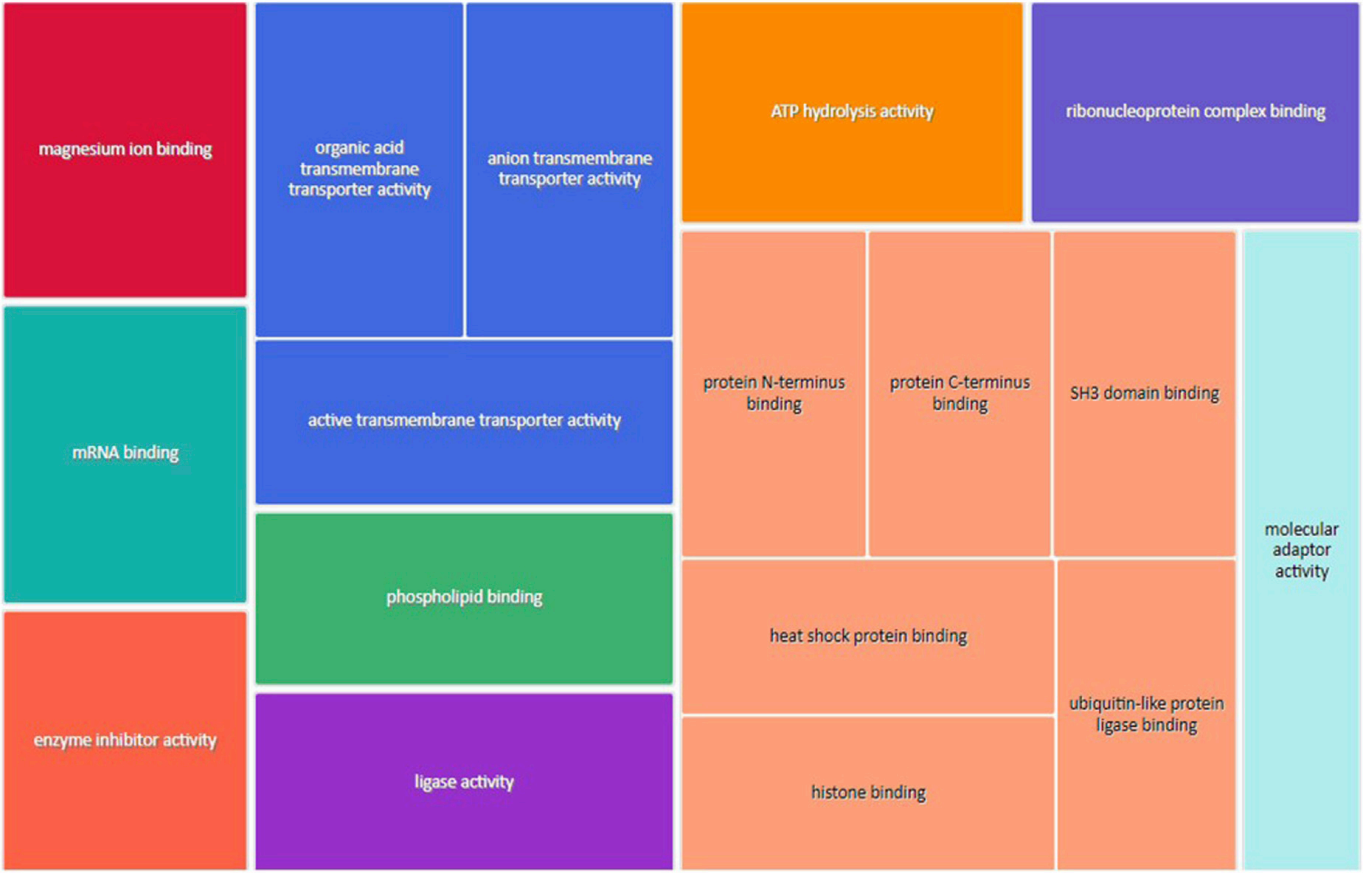
Treemap of GO molecular function categories upregulated in male placentas of obese dams. REVIGO was used to remove redundant GO terms and join the cluster representatives (single rectangles) into superclusters (represented by different colors). Each rectangle’s size reflects the GO term’s false discovery rate (FDR) value (larger for lower FDR), considering a FDR <0.05 as the criterion for statistical significance after Benjamini–Hochberg correction for multiple testing.

Figure 3 shows the most represented non-redundant GO biological process categories upregulated in male placentas of obese dams were signal transduction by a p53 class mediator, positive regulation of the catabolic process, negative regulation of the immune system process, I-kappa kinase/NF-kappa B signaling, the purine-containing compound metabolic process, negative regulation of intracellular signal transduction, and the ErbB signaling pathway. In addition, the critical cellular component GO terms upregulated in male placentas of obese dams were localized in the mitochondrial matrix, lipid droplet, vesicle membrane, actin cytoskeleton, histone deacetylase complex, transcription regulator complex, transcription repressor complex, chromatin, and early endosome (Figure 4). Furthermore, the GO molecular functions of genes associated with an organic acid transmembrane transporter activity, active transmembrane transporter activity, anion transmembrane transporter activity, ribonucleoprotein complex binding, and ATPase activity were increased in male placentas of obese dams as compared to male control placentas (Figure 5).

### GO enrichment analysis for downregulated genes in male placentas of obese dams

The non-redundant GO biological processes downregulated in male placentas of obese dams were a response to nutrient, the transmembrane receptor protein serine/threonine kinase signaling pathway, mitochondrial gene expression, hormone-mediated signaling pathway, response to leukemia inhibitory factor, NADH dehydrogenase complex assembly, and regulation of gene expression, epigenetic (Supplementary Figure S2). In addition, many downregulated genes in male placentas of obese dams were located in the heterochromatin, endoplasmic reticulum-Golgi intermediate compartment, vesicle membrane, recycling endosome, oxidoreductase complex, and nuclear periphery (Supplementary Figure S3). Moreover, the GO molecular function of genes associated with the DNA-binding transcription repressor activity, RNA polymerase II-specific transcription, phospholipid binding, peptide receptor activity, metallopeptidase activity, phospholipid binding, active transmembrane transporter activity, hormone receptor binding, and ubiquitin-like protein binding (Supplementary Figure S4).

### GO enrichment analysis for upregulated genes in female placentas of obese dams

Supplementary Figure S5 shows the most represented biological process categories upregulated in female placentas of obese dams were the cytoplasmic translation, proteasomal protein catabolic process, response to temperature stimulus, response to acid chemical, synaptic vesicle cycle, and protein import. Furthermore, most of the upregulated genes in the female placentas of obese dams were predominantly localized to the outer membrane, coated vesicle, lytic vacuole, condensed chromosome, endoplasmic reticulum membrane, organelle inner membrane, and cell projection membrane (Supplementary Figure S6). The GO molecular function terms upregulated in female placentas of obese dams were magnesium ion binding, structural constituent of the ribosome, kinase regulator activity, enzyme inhibitor activity, inorganic cation transmembrane transporter activity, chromatin DNA binding, and single-stranded RNA binding (Supplementary Figure S7).

### GO enrichment analysis for downregulated genes in female placentas of obese dams

The most enriched non-reductant GO biological functions downregulated in female placentas of obese dams were associated with the muscle system process, chromatin organization, peptidyl serine modification, translational initiation, muscle cell differentiation, sensory system development, regulation of embryonic development, and regulation of the mRNA metabolic process (Supplementary Figure S8). In addition, most downregulated genes in the female placentas of obese dams were predominantly localized to the external side of the plasma membrane, clathrin-coated pit, basolateral plasma membrane, extracellular matrix, cell–substrate junction, myelin sheath, nuclear speck, ribosomes, microtubule-associated complex, and endoplasmic reticulum lumen (Supplementary Figure S9). Furthermore, the GO molecular functions downregulated in the placentas of obese dams were associated with single-stranded RNA binding, GTPase activity, endopeptidase activity, hydrolase activity, acting on carbon–nitrogen (but not peptide) bonds, deubiquitinase activity, helicase activity, glycosaminoglycan binding, growth factor binding, histone binding, and histone deacetylase binding (Supplementary Figure S10).

### KEGG pathway enrichment analysis

KEGG pathway analysis for up- and down-regulated genes in both male and female placentas was analyzed using DAVID and GSEA online tools. For upregulated DEGs in male placentas of obese dams, 10 and 27 KEGG pathways were enriched according to GSEA and DAVID, respectively (Supplementary Tables S6, S7). As shown in Supplementary Figure S11, the GSEA and DAVID tool analyses identified eight common upregulated KEGG pathways in male placentas of obese dams: inositol phosphate metabolism, tight junction, adipocytokine, PPAR, phosphatidylinositol, glycosphingolipid biosynthesis–ganglio series, the T-cell receptor, and the Wnt signaling pathway. For downregulated DEGs in male placentas of obese dams, 13 and 36 KEGG pathways were enriched according to GSEA and DAVID analyses, respectively (Supplementary Tables S8, S9). The 12 common downregulated KEGG pathways in male placentas of obese dams identified by GSEA and DAVID analyses were protein export, DNA replication, aminoacyl-tRNA biosynthesis, purine metabolism, pyrimidine metabolism, oxidative phosphorylation, Huntington’s disease, Parkinson’s disease, RNA polymerase, ubiquitin-mediated proteolysis, Alzheimer’s disease, glycolysis, and gluconeogenesis (Supplementary Figure 12).

KEGG pathway analysis using DAVID and GSEA identified 10 and 18 pathways, respectively, that were enriched with upregulated DEGs in female placentas of obese dams (Supplementary Tables S10, S11). The six KEGG pathways (Supplementary Figure S13) that were commonly enriched in GSEA and DAVID analyses were MAPK signaling, Toll-like receptor signaling, homologous recombination, neurotrophin signaling, GnRH signaling, and T-cell receptor signaling. Supplementary Tables S12, S13 show the list of enriched KEGG pathways downregulated in female placentas of obese dams. The GSEA and DAVID analyses identified 7 and 16 KEGG pathways, respectively. Among these pathways, endocytosis, cysteine and methionine metabolism, pyrimidine metabolism, and ubiquitin-mediated proteolysis were common in both analyses (Supplementary Figure S14). Only one KEGG pathway was found to be enriched (FDR<0.01) with downregulated DEGs.

Maternal obesity alters transcriptomic pathways related to mitochondrial dysfunction, oxidative phosphorylation, and protein ubiquitination in male placentas of obese dams.

IPA revealed the differential regulation of 154 significant (*p* < 0.05) canonical pathways in male placentas in response to maternal obesity, including oxidative phosphorylation, ceramide signaling, protein ubiquitination, PKCθ signaling in T lymphocytes, sirtuin, Rac, PDGF signaling, CD27 signaling in lymphocytes, production of nitric oxide and reactive oxygen species in macrophages, p70S6k, angiopoietin, and B-cell receptor signaling (Supplementary Table S14).

The expression of many genes in the placental p21-activated kinases (*Pak*, *p* = 1.418 E-02) and *Rac* (*p* = 1.14 E-02) signaling pathways were upregulated in response to maternal obesity in male placentas. PAK are a family of critical effectors of *Rac1* and *Cdc42*. They regulate various cellular functions, such as cytoskeleton dynamics, cell movement and migration, cell proliferation and differentiation, and gene expression. Sixteen of 109 genes in this pathway were differentially expressed, including phosphatidylinositol 3-kinase catalytic subunit type 3 (*Pik3c3*), myosin light chain 1 (*Myl1*), myosin light chain, phosphorylatable, fast skeletal muscle (*Mylpf*), mitogen-activated protein kinase 8 (*Mapk8*), myosin light polypeptide 6 (*Myl6*), P21 (Rac1)-activated kinase 6 (*Pak6*), and RAP2B, a member of the RAS oncogene family (*Rap2b*)*.* In addition, members of *Rac1* signaling pathway genes such as RAS homology family A (*Rhoa*) and CD44 (*Cd44*) were all significantly upregulated in the obese group.

The 70-kDa ribosomal protein S6 kinase (*p70S6K*) signaling pathway (*p* = 1.84E-03) is known to promote protein synthesis and cell growth. Nineteen of 113 genes in this pathway were differentially expressed. Genes such as ribosomal protein S6 kinase B1 (*Rps6kb1*), casein kinase 2 beta (*Csnk2b*), insulin receptor substrate 2 (*Irs-2*), and protein kinase C Zeta (*Prkcz*) were significantly upregulated in the obese group as compared to control.

A large number of genes, 108 of 171 genes, in the mitochondrial functional pathway (*p* = 1.86E-05) were downregulated in male placentas of obese dams. Genes such as ATP synthase F1 subunit beta (*Atp5b*)*,* cytochrome c oxidase Subunit 6B1 (*Cox6b1*), NADH dehydrogenase [ubiquinone] 1 alpha subcomplex subunit 3 (*Ndufa3*), NADH dehydrogenase [ubiquinone] 1 alpha subcomplex subunit 7 (*Ndufa7*), cytochrome c oxidase (*Cox15*), NADH: ubiquinone oxidoreductase subunit A11 (*Ndufa11*), and ATP synthase F1 subunit beta (Atp*5f1b*) were all significantly downregulated in the obese group.

Maternal obesity alters transcriptomic pathways related to triacylglycerol biosynthesis and cardiac hypertrophy but not oxidative phosphorylation pathways in female placentas of obese dams.

The IPA analysis of DEGs in female placentas revealed that 63 significant (*p* < 0.05) canonical pathways were differentially regulated in response to maternal obesity, including sirtuin signaling, triacylglycerol biosynthesis, and phosphatidylglycerol biosynthesis II (Supplementary Table S15).

Eight of 47 genes were differentially expressed in the triacylglycerol biosynthesis pathway (*p* = 1.27 E-03). The pathway was predicted to be downregulated due to expression of genes such as porcupine O-acyltransferase (*Porcn*), tafazzin (*Taz*), lysocardiolipin acyltransferase 1 (*Lclat1*), phospholipid phosphatase 5 (*Plpp5*), dihydrolipoamide branched chain transacylase E2 (*Dbt*), lysophosphatidylcholine acyltransferase 1 (*Lpcat1*), and lycerol-3-phosphate acyltransferase 4 (*Gpat4*), which were all significantly decreased in female placentas of obese dams.

The expression of 22 out of 242 genes in the cardiac hypertrophy signaling pathway was increased in female placentas of obese dams compared to controls (*p* = 1.98 E-03). Genes such as myosin light chain 1 (*Myl1*), myosin light chain, phosphorylatable, fast skeletal muscle (*Mylpf*), activating transcription factor 2 (*Atf2*), myocyte enhancer factor 2C (*Mef2c*), RAS homolog family member D (*Rhod*), calcium voltage-gated channel subunit alpha1 D (*Cacna1d*), and mitogen-activated protein kinase 14/8/6/3 (*Mapk14/8/6/3*) were significantly upregulated in response to maternal obesity*.*

### Maternal obesity alters upstream regulators in a fetal sex-dependent manner

IPA identified the differential expression of multiple upstream transcriptional regulators that may explain changes in gene expression and illuminate the biological activities occurring in the tissue of interest including serine/threonine-protein kinase/endoribonuclease inositol-requiring enzyme 1α (*ERN1*), interleukin 4 (*IL-4*), activating transcription factor 6 (*ATF6*), thrombospondin-4 (*THSB4*), and hypoxia-inducible factor 1-alpha (*HIF-1 alpha*), which were decreased in both male and female placentas of obese dams. Conversely, the upstream regulators that were activated in both male and female placentas of the obese group were miR-124-3p, miR-1-3p, miR-16-5p, mitogen-activated protein kinase 7 (*MAPK7*), and tumor necrosis factor (ligand) superfamily, member 10 (*TNFSF10*). Interestingly, interleukin-2 (*IL-2*) and peroxisome proliferator-activated receptor gamma coactivator 1-alpha (*PGC-1α*) pathways were activated in male placentas but inhibited in female placentas of obese dams (Supplementary Figure S15).

### Effect of maternal obesity on the expression of proteins involved in the mitochondrial function

**FIGURE 6:**
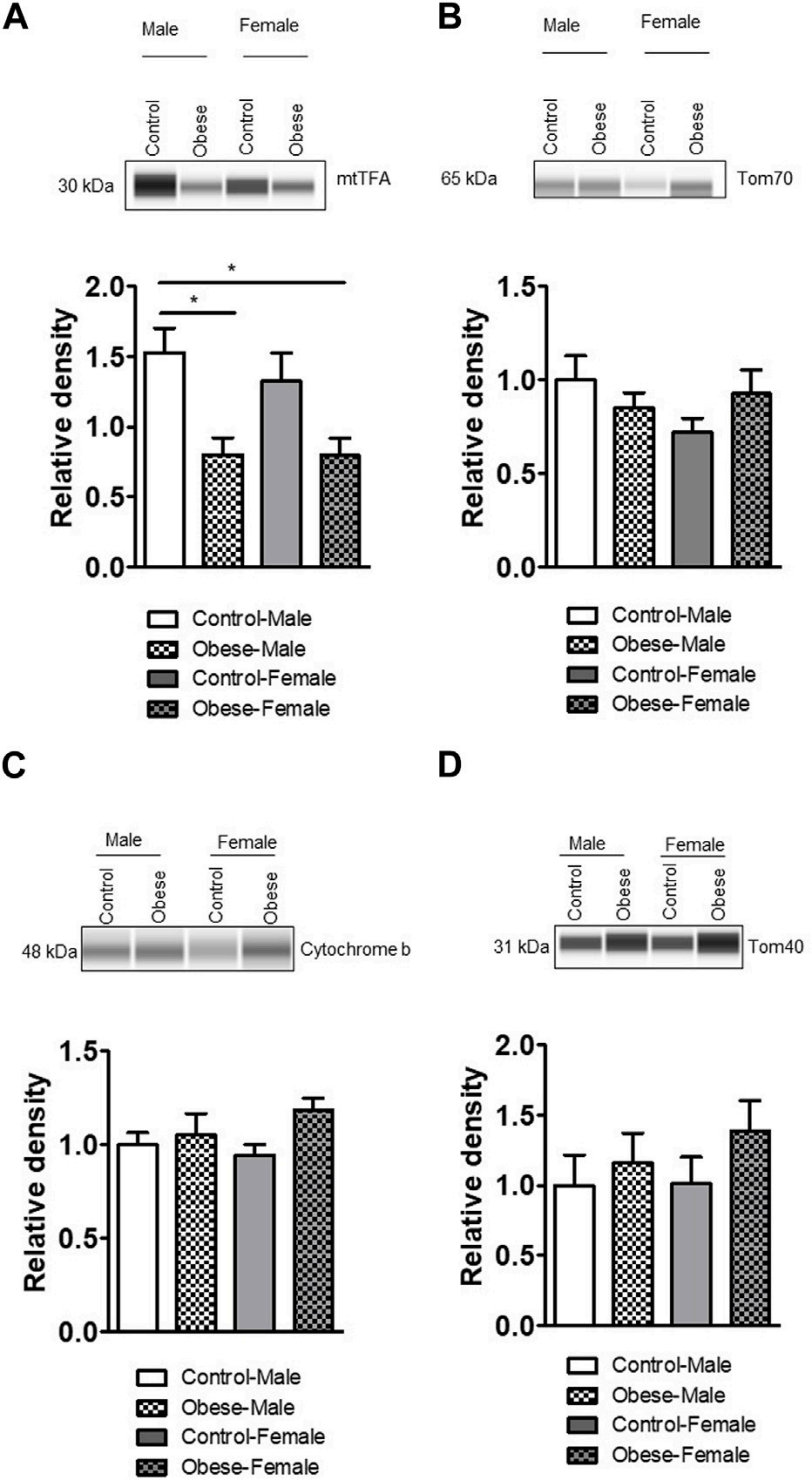
Effect of maternal obesity on the expression of placental mtTFA, Tom70, cytochrome b, and Tom40 in pregnant mice. **(A–D)** Protein expression of mtTFA, Tom70, cytochrome b, and Tom40 in the placental homogenates of obese and control groups (n = 7/each group). A representative Western blot is shown. Data are from a representative experiment, and similar results were obtained from six other experiments. Values are means +SEM.; *p < 0.05 vs. control by one-way ANOVA with the Tukey–Kramer multiple comparison *post hoc* test.

**FIGURE 7:**
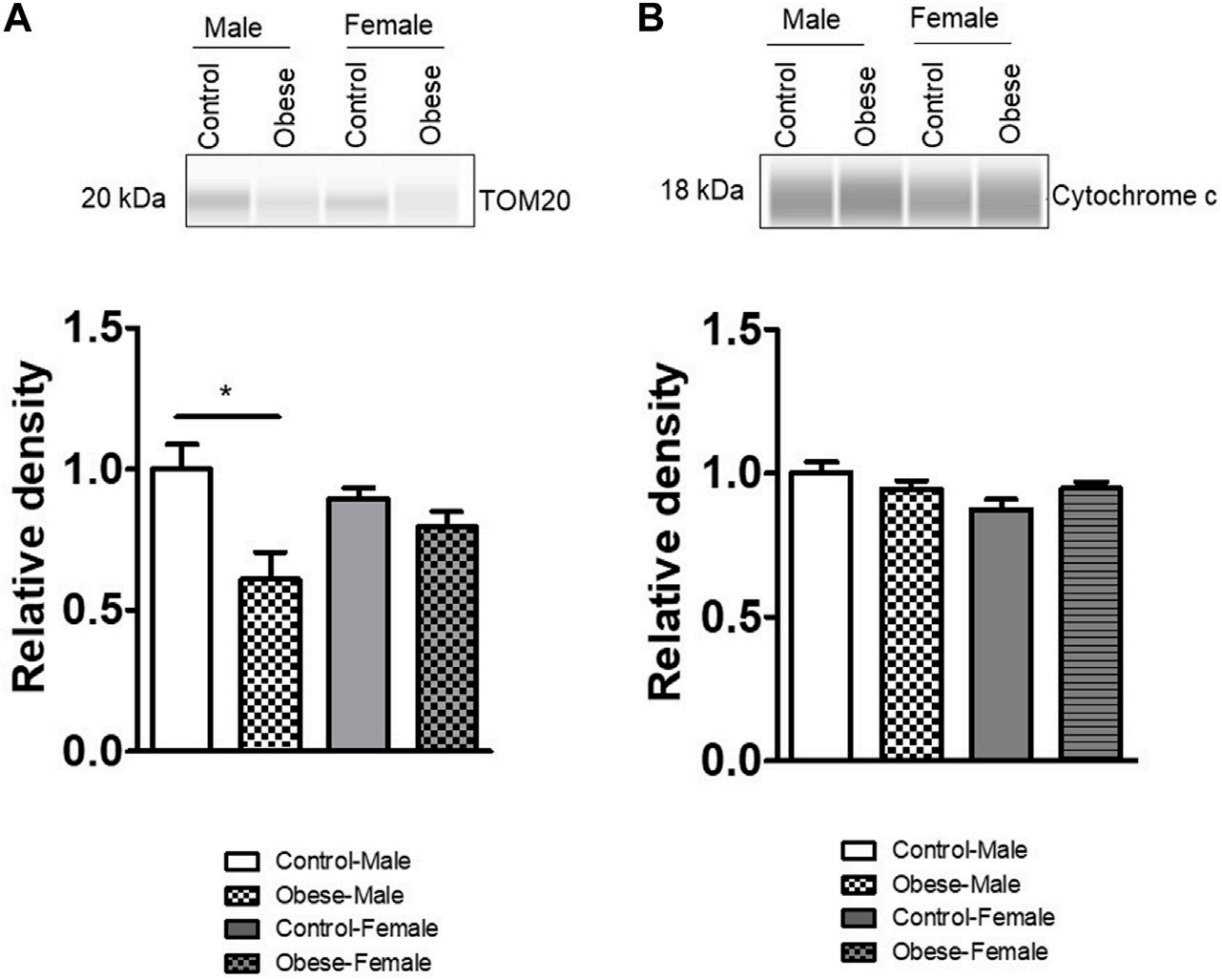
Effect of maternal obesity on the placental expression of Tom20 and cytochrome c in pregnant mice. **(A,B)** Protein expression of Tom20 and cytochrome c in the placental homogenates of obese and control groups (n = 7/each group). A representative Western blot is shown. Data are from a representative experiment, and similar results were obtained from six other experiments. *p < 0.05 vs. control by one-way ANOVA with the Tukey–Kramer multiple comparison *post hoc* test.

We measured the protein expression of mtTFA (mitochondrial transcription factor A), Tom (Translocase of outer membrane)-70, cytochrome b, Tom40, Tom20, and cytochrome c in the placental homogenates of obese and control groups (Figures 6, 7). The protein expression levels of mtTFA and Tom20 were significantly reduced in male placentas of obese dams as compared to the control group, whereas maternal obesity did not affect the mtTFA and Tom20 expression levels in female placentas. In contrast, the protein expression levels of Tom70, Tom40, cytochrome b, and cytochrome c were comparable between groups.

### Effect of maternal obesity and birth weight on mitochondrial electron transport chain complex expression in the human placenta

**FIGURE 8:**
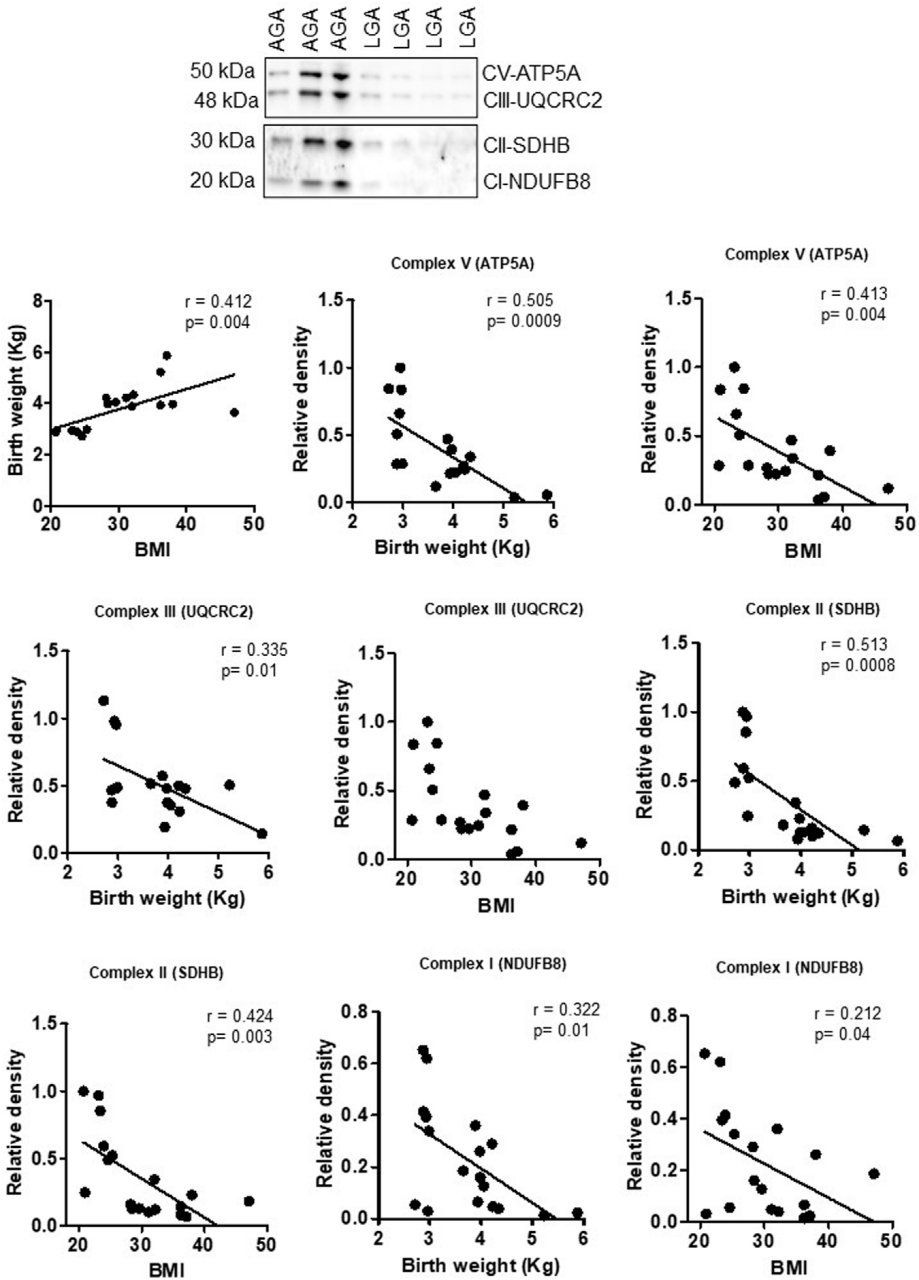
Correlation between maternal BMI, birth weight, and placental mitochondrial, ETC complex (V, III, II, and I) protein expression in male placentas of control (AGA, appropriate-for-gestational-age) and LGA (large-for-gestational-age) pregnancy. r = Pearson’s correlation coefficient, n = 7–11/each group. Placental mitochondrial, ETC complex subunit protein expression in relation to BMI and birth weight. A representative Western blot for mitochondrial, ETC complex subunits (complex V- ATP5F1A, ATP synthase F1 subunit alpha; complex III- UQCRC2, ubiquinol–cytochrome C reductase core protein 2; complex II- SDHB, succinate dehydrogenase complex iron sulfur subunit B; complex I- NDUFB8, NADH-ubiquinone oxidoreductase subunit B8) in homogenates of placentas from pregnancies with varying maternal BMI and birth weights. There was no significant correlation between BMI and mitochondrial, ETC complex subunit III.

**FIGURE 9:**
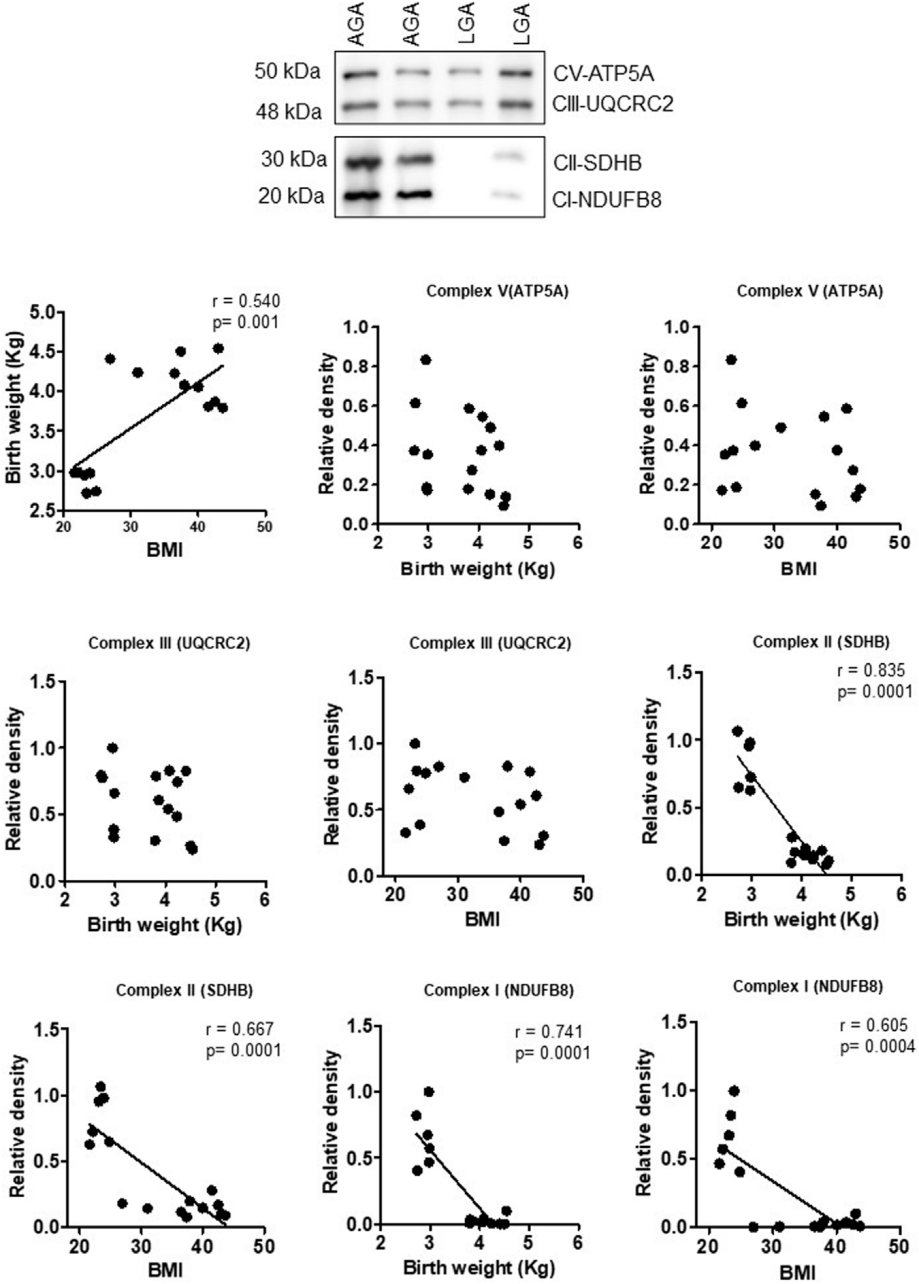
Correlation between maternal BMI, birth weight, and placental mitochondrial, ETC complex (V, III, II, and I) protein expression in female placentas of control (AGA, appropriate-for-gestational-age) and LGA (large-for-gestational-age) pregnancy. r = Pearson’s correlation coefficient, n = 6–10/each group. Placental mitochondrial, ETC complex subunit protein expression in relation to BMI and birth weight. A representative Western blot for mitochondrial, ETC complex subunits (complex V- ATP5F1A, ATP synthase F1 subunit alpha; complex III- UQCRC2, ubiquinol–cytochrome C reductase core protein 2; complex II- SDHB, succinate dehydrogenase complex iron sulfur subunit B; complex I- NDUFB8, NADH-ubiquinone oxidoreductase subunit B8) in homogenates of placentas from pregnancies with varying maternal BMI and birth weights. There was no significant correlation between BMI or birth weight and mitochondrial, ETC complex subunits II and I.

To explore the clinical significance of our findings in a mouse model of maternal obesity, we investigated the relationships among maternal obesity, birth weight, and the protein expression of placental mitochondrial electron transport chain (ETC) complexes. Even in this small sample size, maternal BMI was positively correlated with birth weight in both sexes (Figures 8, 9). Figure 8 shows that the protein expression levels of ETC complexes V, II, and I in male placentas were negatively correlated with maternal BMI and birth weight. Male placental, ETC complex III expression was negatively correlated with birth weight but did not correlate with maternal BMI. In contrast (Figure 9), there was no significant relationship between maternal BMI or birth weight and the protein expression of placental mitochondrial, ETC complexes III and V in pregnancies with a female infant. On the other hand, ETC complex I and II expression in the female placenta was negatively correlated with maternal BMI and birth weight.

## Discussion

Using a mouse model of high-fat and high-sugar diet-induced maternal obesity with fetal overgrowth, we report that maternal obesity impacts the placental transcriptome in a sex-dependent manner. RNA-seq analysis demonstrated that a total of 2287 genes were differentially expressed in the placentas of obese dams. We found a significant impact of maternal obesity on genes related to mitochondrial function in male placentas of obese dams, and we provided evidence that the findings in mice have clinical relevance by demonstrating that human placental mitochondrial, ETC expression was downregulated and negatively correlated with maternal pre-pregnancy BMI and birth weights in male infants.

Top downregulated genes (*Nudt*13 and *Gsta*3) in the male placentas of obese dams were associated with mitochondrial dysfunction. *Nudt13* is a NAD(P)H pyrophosphatase that uses mitochondrial NADH and/or NADPH as a substrate. *Nudt13* is an important regulator of the redox state of the cell and may act as a redox sensor for transcriptional control in cells (Abdelraheim et al., 2017).

We found a significant reduction in the transcript levels of genes encoding several mitochondrial proteins such as *Ndufa7, Ndufa9, Ndufa10, Ndufa4l2*, and *Ndufa11* in male placentas of obese dams. In addition, we demonstrated that the protein expression of TOM20 and mtTFA was lower in placentas from male fetuses of obese dams. TOM20 is anchored to the outer membrane as a peripheral subunit of the TOM40 complex (Yamamoto et al., 2011). Recent evidence indicates that the defective mitochondrial import due to the decreased expression of TOM20 contributes to complex I-mediated mitochondrial dysfunction in the brain and the development of neurogenerative disease (Franco-Iborra et al., 2018). Furthermore, reduction in the mitochondrial protein import activity was linked to reduced protein levels of OXPHOS subunits (Schmidt et al., 2010). mtTFA is an essential high-mobility protein that enhances mtDNA transcription, replication, and synthesis of subunits encoded in mtDNA (Alam et al., 2003). The knockout of the mtTFA gene in mice results in the reduction of mtDNA and embryonic lethality (Larsson et al., 1998). Placental mtTFA expression has also been reported to be decreased in pre-eclampsia (Vishnyakova et al., 2016). Our data suggest that exposure to maternal obesity in mice leads to a state of mitochondrial dysfunction in the placentas of male fetuses.

We provided evidence that human placental mitochondrial, ETC complex (V, III, II, and I) expression was downregulated and negatively correlated with maternal pre-pregnancy BMI and birth weight in male infants. Obesity has been reported to be associated with a decrease in the mtDNA copy number, mitochondrial mass, mitochondrial ETC complexes, and mitochondrial activity/biogenesis in white adipose tissue in mouse models and in skeletal muscle of obese men and women (Kelley et al., 2002). Mele et al. showed significant reduction in mitochondrial respiration in cultured primary trophoblasts isolated from pregnancies complicated by maternal obesity (Mele et al., 2014). Thus, reduction in the expression of placental mitochondrial-associated genes in pregnancies complicated by maternal obesity may compromise the placental function and potentially underlying the increased susceptibility of male fetuses of obese mothers to developmental programming in later life (Mele et al., 2014). In addition, maternal obesity stimulates lipotoxicity and increases the activation of inflammatory NF-κB and JNK signaling in the placenta (Liang et al., 2018). NF-κB controls mitochondrial dynamics (Albensi, 2019). Thus, upregulation of the NF-κB signaling pathway may contribute to decreased oxidative phosphorylation reported in the placentas of males in maternal obesity. Alternatively, the downregulation of placental mitochondrial function in male fetuses of obese mice and in women may represent an adaptive response to intermittent hyperglycemia common in maternal obesity, which could be associated with a shift of placental energy production from oxidative phosphorylation to glycolysis.

Other molecular pathways that were downregulated in the male placentas of obese dams are involved in neurodegenerative disorders, namely, Huntington disease and Alzheimer disease pathways. Recent studies indicate that there might be common developmental pathways between the brain and placenta (Burton and Jauniaux, 2018; Maslen, 2018). Moreover, human placenta-derived stem cells differentiate into neural progenitor cells *in vitro*, and the placenta secretes a wide array of neurohormones that regulate fetal brain development (Rosenfeld, 2021). Recent human observational studies suggest that the odds for adverse neurodevelopmental outcome were 17% higher among children of mothers who were overweight or obese during pregnancy (Sanchez et al., 2018). In addition, muscle system processes were among the most enriched downregulated GO biological function in female placentas of obese dams. Although of unclear significance, it is intriguing to note that Shelly and coworkers reported that skeletal muscle signaling and mitochondrial complex II–II linked activity are altered in adult offspring of obese mice (Shelley et al., 2009).

Based on our RNA-seq data, the ceramide signaling pathway is predicted to be upregulated in the male placentas of obese dams. Ceramide is a sphingolipid that functions as an important second messenger known to affect mitochondria, altering both morphology and physiology. The activation of ceramide signaling decreases the mitochondrial oxygen consumption in myotubes (Summers et al., 2019). Thus, it is possible that the upregulation of the ceramide signaling pathway contributes to decreased oxidative phosphorylation reported in placentas of males in maternal obesity (Bucher et al., 2021).

Thermogenesis is another downregulated placental molecular pathway in male fetuses of obese dams. A previous study reported that maternal obesity in mice reduced the expression of genes involved in the thermogenesis of brown adipose tissue (BAT) in offspring (Zhang et al., 2021). Furthermore, decreased thermogenesis in the BAT of fetal mice of obese dams was associated with elevated BAT lipid droplets and reduced mitochondrial DNA and mitochondrial biogenesis markers (Lettieri Barbato et al., 2015). It is therefore plausible that lower oxidative phosphorylation in male placentas of obese dams was caused by the downregulation of the thermogenesis signaling system. Interestingly, decreased thermogenesis was recently highlighted as a possible mechanism underlying altered energy balance in offspring of obese mice (Maragkoudaki et al., 2020).

A top downregulated placental gene in females of obese dams was associated with water transport. Aquaporin 5 (*Aqp5*) belongs to a family of membrane channel proteins that facilitate bulk water transport. *Aqp5* may be involved in energy metabolism, or may be crucial in the clearance of excess lactate in the placental extracellular space (Badaut and Regli, 2004).

Male and female fetuses respond differently to maternal obesity resulting in different risk profiles for developing metabolic diseases later in life (Myatt and Maloyan, 2016). For example, in our mouse model of obesity, male offspring develop obesity, glucose intolerance, and insulin resistance at 3 months of age, whereas the metabolic phenotype in female offspring is much less severe at this age (Paulsen et al., 2019). There is also evidence that the female and male placentas are functionally different with distinct responses to an adverse intrauterine environment such as maternal obesity. Reduced placental mitochondrial function may contribute to impaired glucose metabolism and cardiac dysfunction reported in 3-month-old male offspring born to obese dams (Paulsen et al., 2019; Vaughan et al., 2020).

We also report that the sirtuin signaling pathway was upregulated in the male placentas of obese dams. Sirtuin1 (SIRT1), a class III histone deacetylase, regulates various physiological and pathophysiological processes, including cellular inflammation and metabolism. Recent studies suggest that maternal inflammation is associated with increased placental SIRT1 expression (Park et al., 2020). Furthermore, SIRT1-mediated inflammatory processes may result in placental dysfunction and the release of inflammatory mediators (Ilekis et al., 2016).

In healthy human pregnancies, sex differences in placental size and gene expression are evident throughout the gestation (Buckberry et al., 2014). This is believed to play a role in determining sex differences in intrauterine growth that are apparent from the early stages of development. For example, as early as the pre-implantation stage, male embryos grow faster, demonstrating fundamental differences in growth and metabolism between the sexes (Mittwoch, 1993). In addition, based on the placental weight required to sustain a fetus (Eriksson et al., 2010), male placentas may be more efficient than female placentas.

Studies in rodents suggest that changes in placental morphology and placental inflammation in obese dams are more pronounced in male than female offspring (Kim et al., 2014). In women, Wang et al. demonstrated that fuel utilization in trophoblasts for mitochondrial respiration in human pregnancies complicated by obesity and GDM is sexually dimorphic (Wang et al., 2019). Specifically, as compared to female offspring, male placentas in maternal obesity and GDM were found to have decreased fuel flexibility with an increased dependency on glucose and limited ability to use glutamine for oxidative phosphorylation (Wang et al., 2019).

Evans and Myatt (2017) demonstrated that in lean women, the male placentas have greater antioxidant defense. However, this protection is lost with maternal obesity, perhaps contributing to the increased incidence of adverse outcomes in male infants (Peacock et al., 2012; Evans and Myatt, 2017). We observed that placental GST (glutathione-S-transferase) gene expression was decreased in males of obese dams. GST is an essential component of the cellular antioxidant defense mechanism that catalyzes the conjugation of reduced glutathione with a large array of xenobiotics and endogenous electrophiles (Yang et al., 2001). GST is also involved in the biosynthesis of leukotrienes, prostaglandins, testosterone, and progesterone, and the degradation of tyrosine (Hayes et al., 2005). The underlying cause for these sexually dimorphic changes in placental gene expression remains to be investigated.

Using the same mouse model of maternal obesity as in the current study, we previously reported the effect of maternal obesity on the fetal liver transcriptome (Kelly et al., 2022). Differentially regulated genes in the liver and placentas in the current study were distinct, with a little overlap. This may reflect that while the placenta is directly exposed to the metabolic changes in the maternal circulation, the fetal liver is not.

The mouse model used in the current study was originally developed to study maternal obesity associated with fetal overgrowth because LGA babies of obese women have a higher risk than appropriate-for-gestational age (AGA) babies to develop cardiovascular diseases later in life. Although obese women are more likely to deliver LGA babies (Yu et al., 2013; Leon-Garcia et al., 2016), the majority of the babies born to obese mothers have a birth weight that is AGA. Importantly, also AGA babies of obese mothers may have increased adiposity and evidence of insulin resistance, predisposing them for cardiometabolic diseases later in life. In the current study, maternal obesity did not alter the fetal/placental ratio. However, maternal obesity in our model causes increased placental glucose and amino acid transport (Rosario et al., 2015), which likely contributes to increased fetal growth. In contrast, using a different mouse model of diet-induced maternal obesity, Lager and coworkers reported normal placental and fetal weights but a lower fetal/placental ratio, suggesting decreased placental efficiency (Lager et al., 2014). It is notable that offspring in this model are programmed for future diseases (Samuelsson et al., 2008; Kirk et al., 2009; Oben et al., 2010; Samuelsson et al., 2010; Reynolds et al., 2013; Samuelsson et al., 2013; Taylor et al., 2014), demonstrating that the link between maternal obesity and offspring cardiometabolic disorders is not limited to infants born large.

In conclusion, our findings have advanced our understanding of the multifaceted effects of maternal obesity on the placental transcriptome that may be crucial to placenta development and function and impacting the developing fetus. We report that maternal obesity with fetal overgrowth differentially regulates the transcriptome in male and female placentas, including genes involved in oxidative phosphorylation. We provide evidence that the findings in mice have clinical relevance by demonstrating that human placental mitochondrial complex expression was downregulated and negatively correlated with maternal pre-pregnancy BMI and birth weight in male infants. This new information could potentially exert efforts to identify new interventions to alleviate the increased risk for poor metabolic health in offspring exposed to maternal obesity.

## Data Availability

The datasets presented in this study can be found in online repositories. The names of the repository/repositories and accession number(s) can be found in the article/Supplementary Material.
