# Supplementary material for: Maternal obesity alters the placental transcriptome in a fetal sex-dependent manner
Source: Front Cell Dev Biol. 2023 Jun 15;11:1178533. doi: 10.3389/fcell.2023.1178533 (PMC10309565; doi:10.3389/fcell.2023.1178533)
Supplement: Supplementary file 2 [file Presentation14.PPTX]

## Slide 1
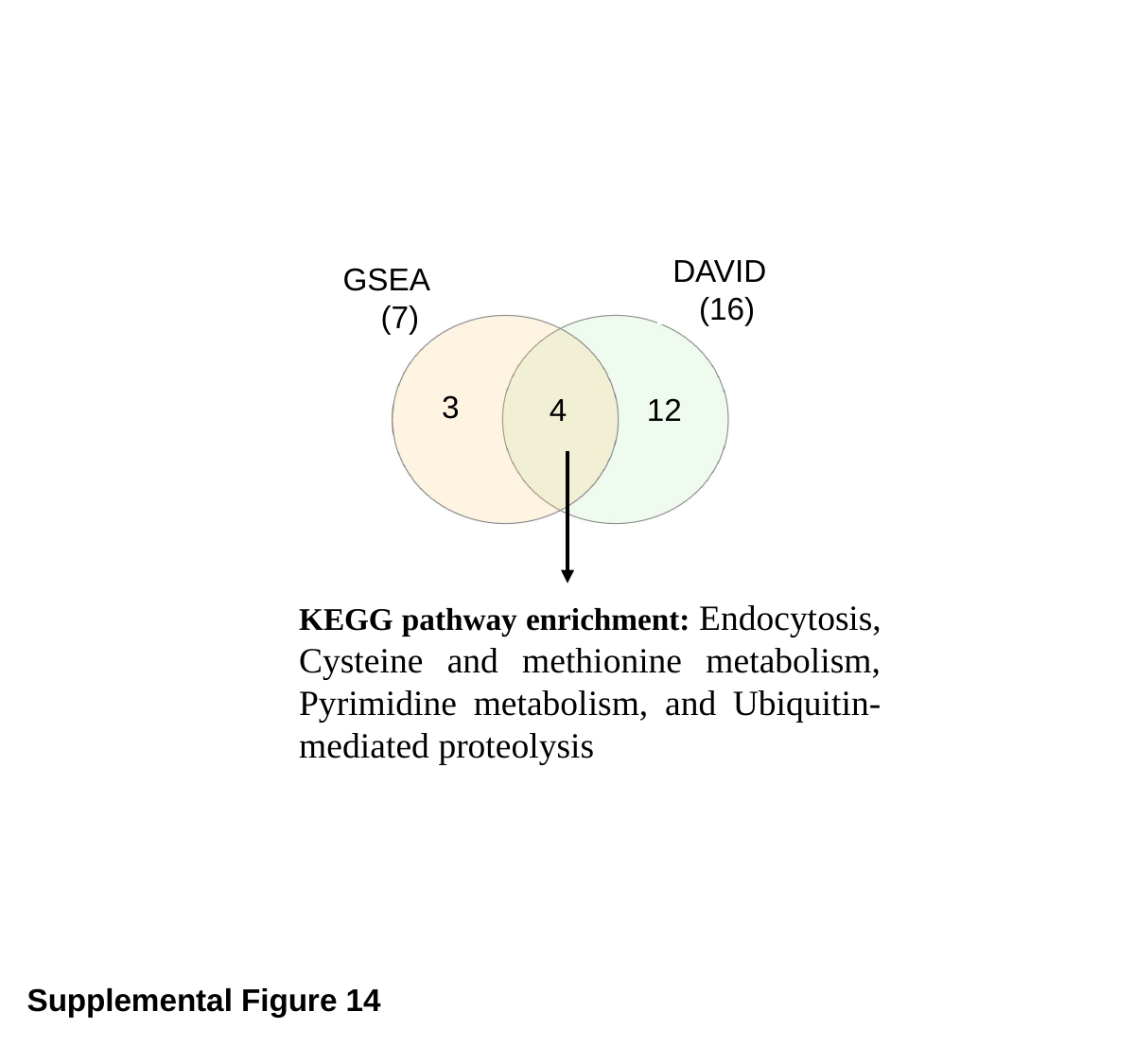

GSEA
 (7)
DAVID
 (16)
3
12
4
KEGG pathway enrichment: Endocytosis, Cysteine and methionine metabolism, Pyrimidine metabolism, and Ubiquitin-mediated proteolysis
Supplemental Figure 14
