# Supplementary figures and images for: Maternal obesity alters the placental transcriptome in a fetal sex-dependent manner
Source: Front Cell Dev Biol. 2023 Jun 15;11:1178533. doi: 10.3389/fcell.2023.1178533 (PMC10309565; doi:10.3389/fcell.2023.1178533)

## Slide 1
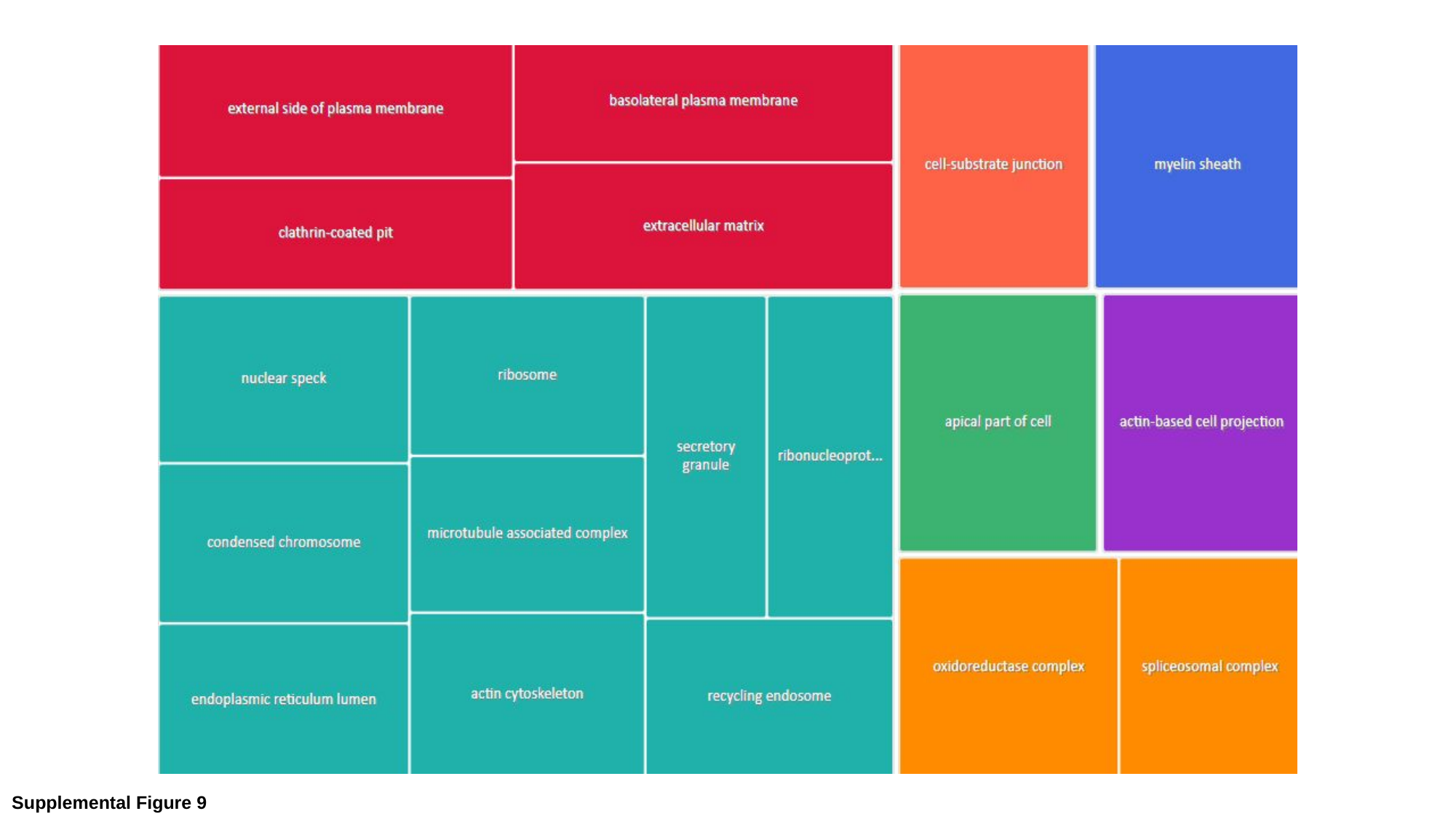

Supplemental Figure 9

Supplement: Supplementary file 4 [file Presentation9.PPTX]

## Slide 1
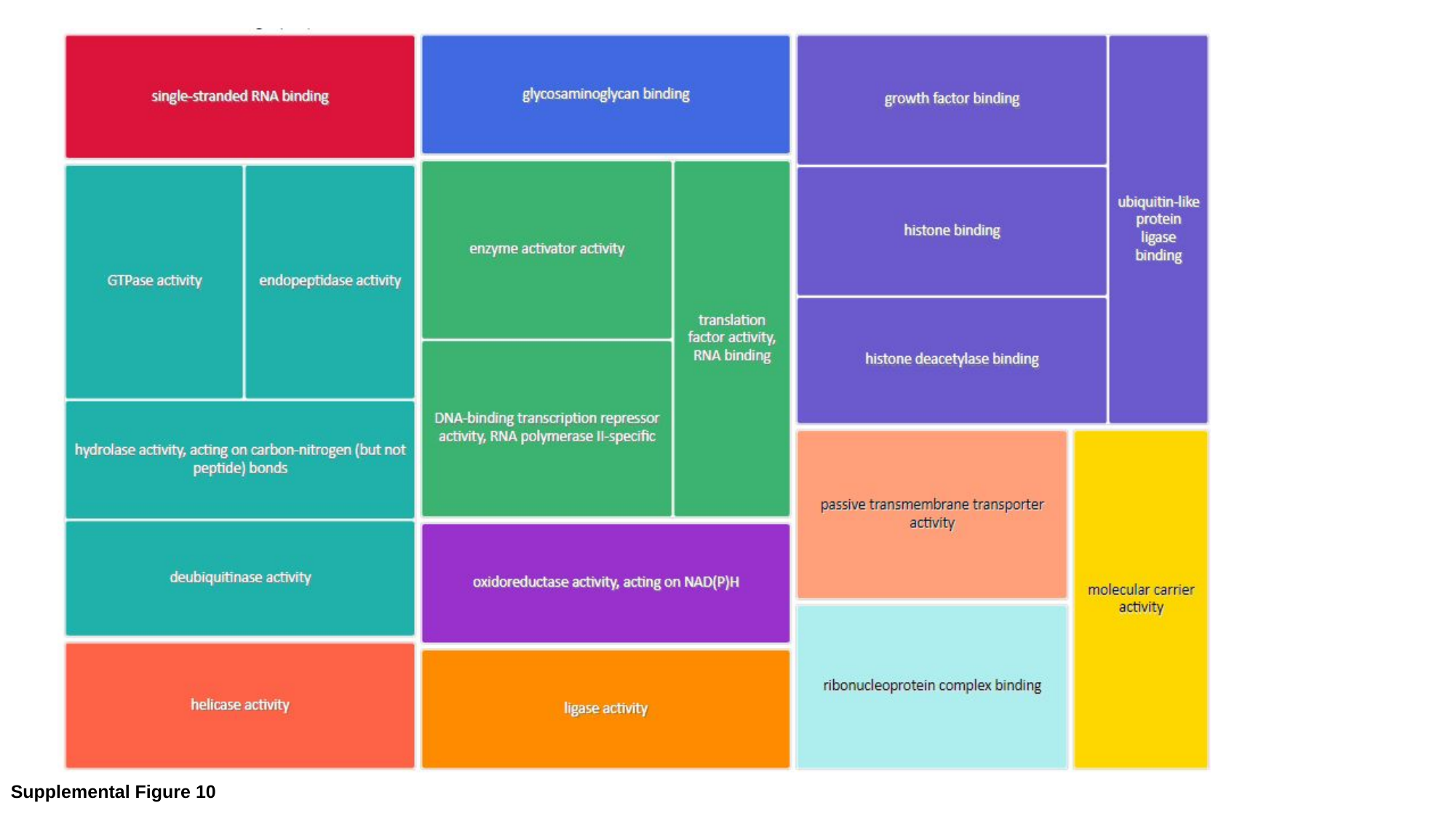

Supplemental Figure 10

Supplement: Supplementary file 6 [file Presentation10.PPTX]

## Slide 1
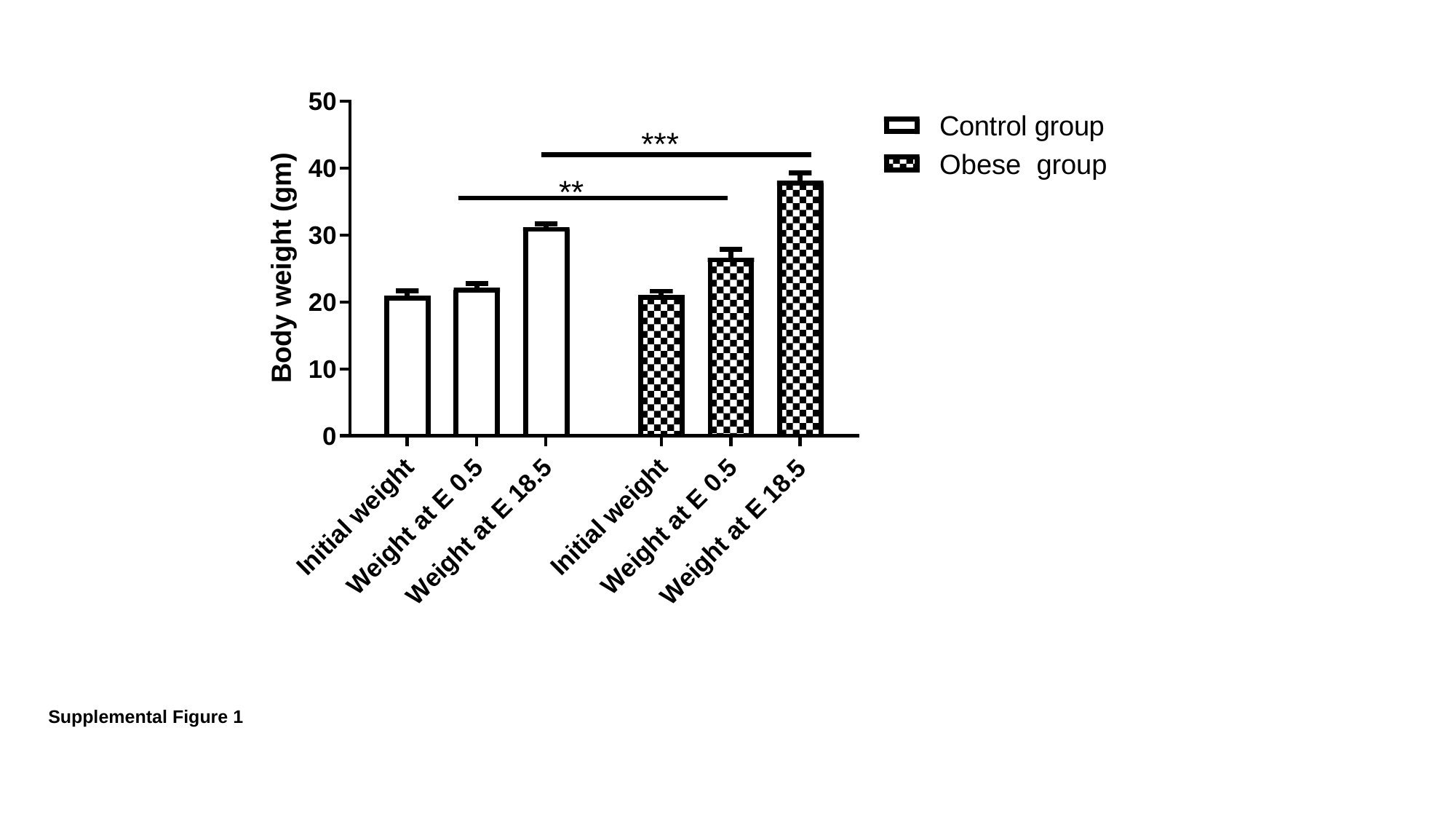

Supplemental Figure 1

Supplement: Supplementary file 7 [file Presentation1.PPTX]

## Slide 1
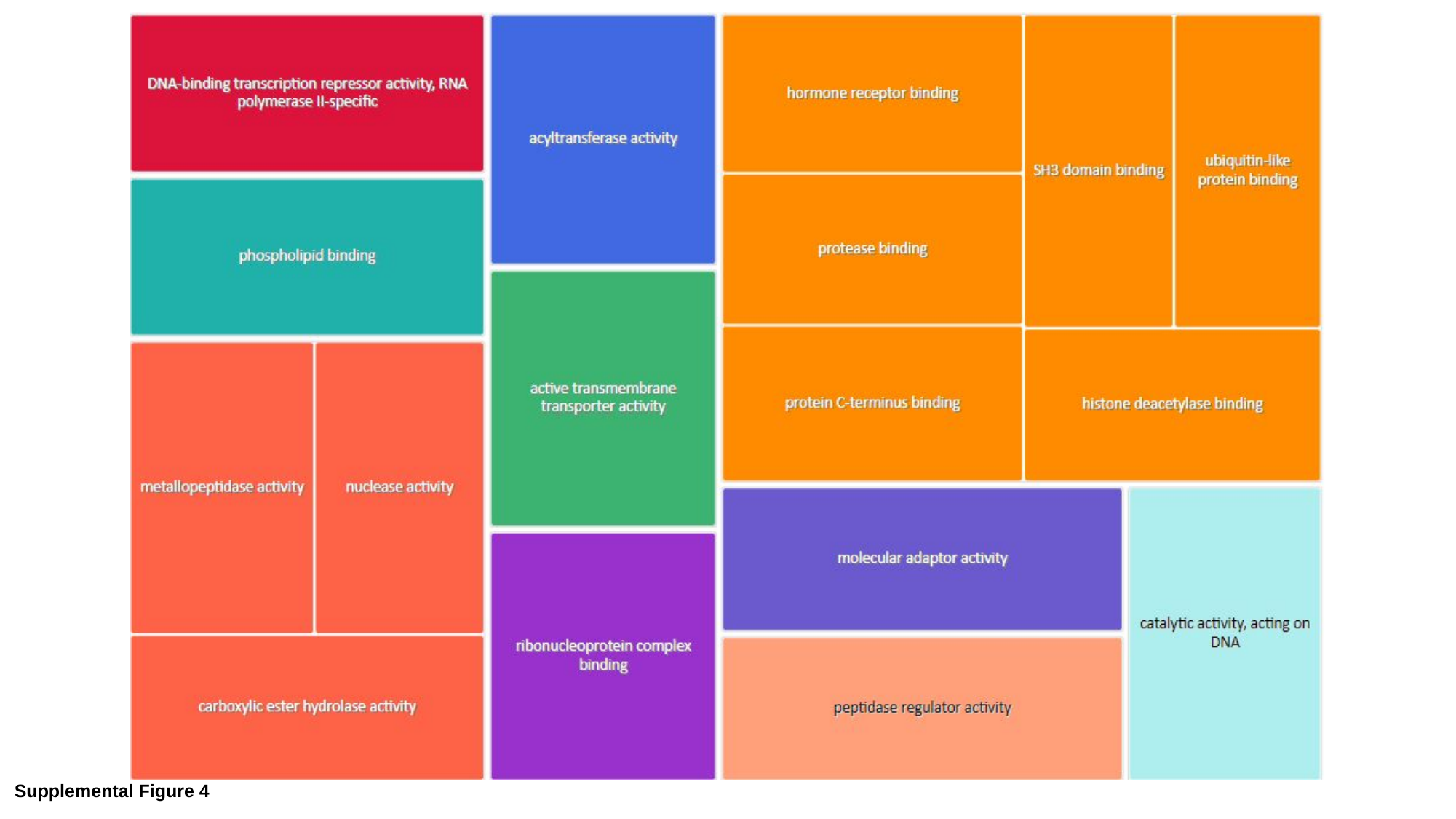

Supplemental Figure 4

Supplement: Supplementary file 13 [file Presentation4.PPTX]

## Slide 1
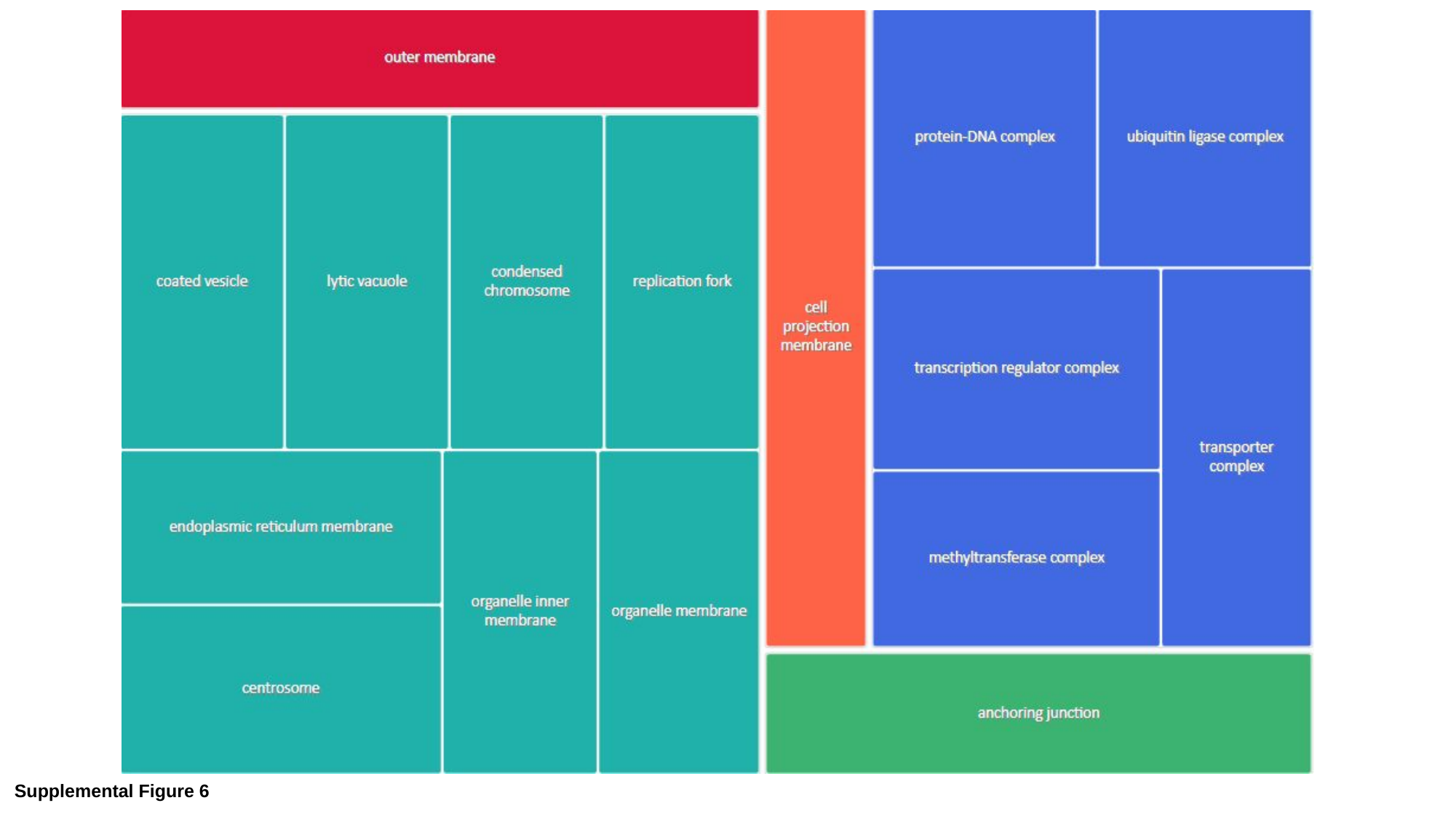

Supplemental Figure 6

Supplement: Supplementary file 15 [file Presentation6.PPTX]

## Slide 1
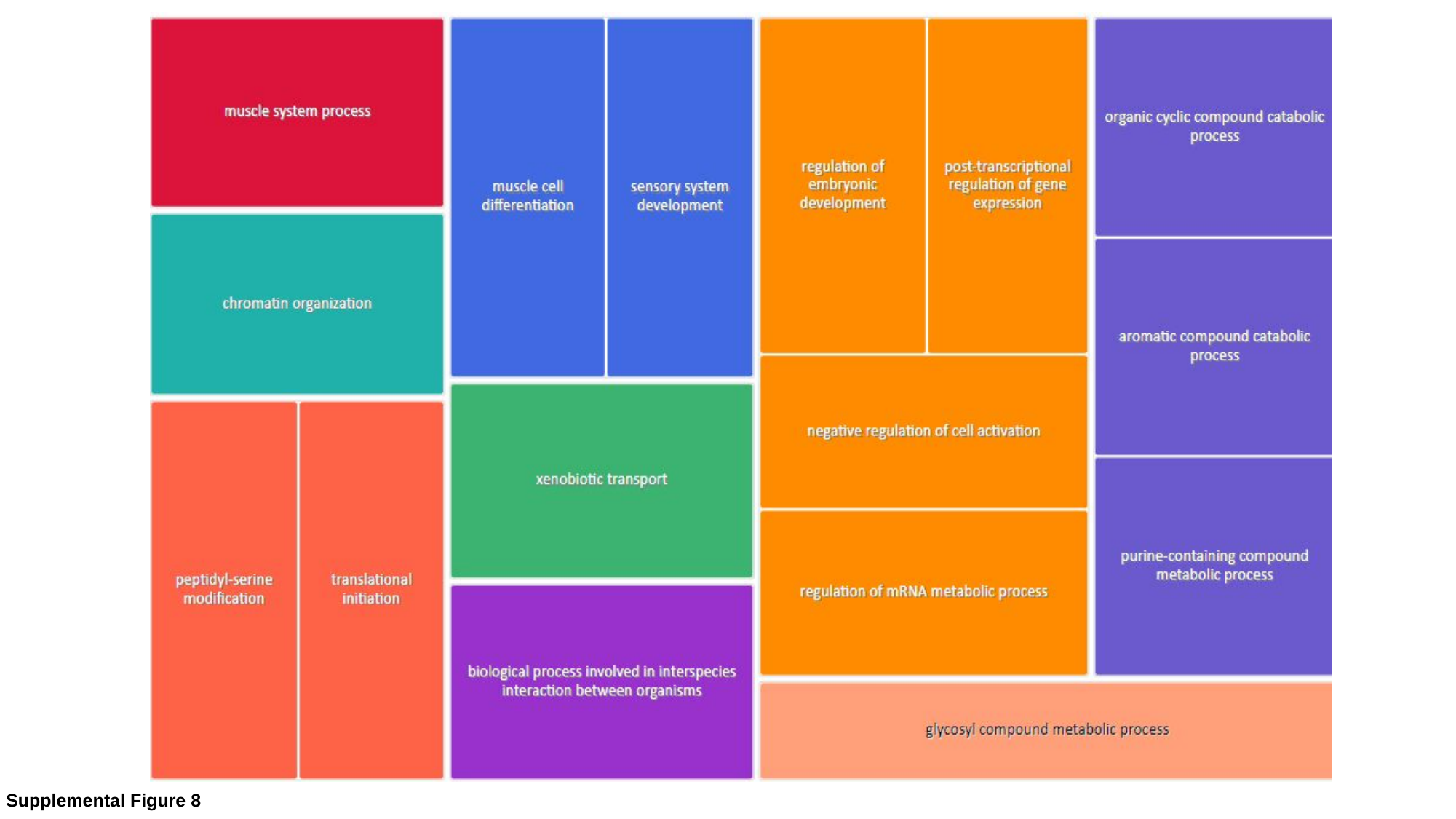

Supplemental Figure 8

Supplement: Supplementary file 19 [file Presentation8.PPTX]

## Slide 1
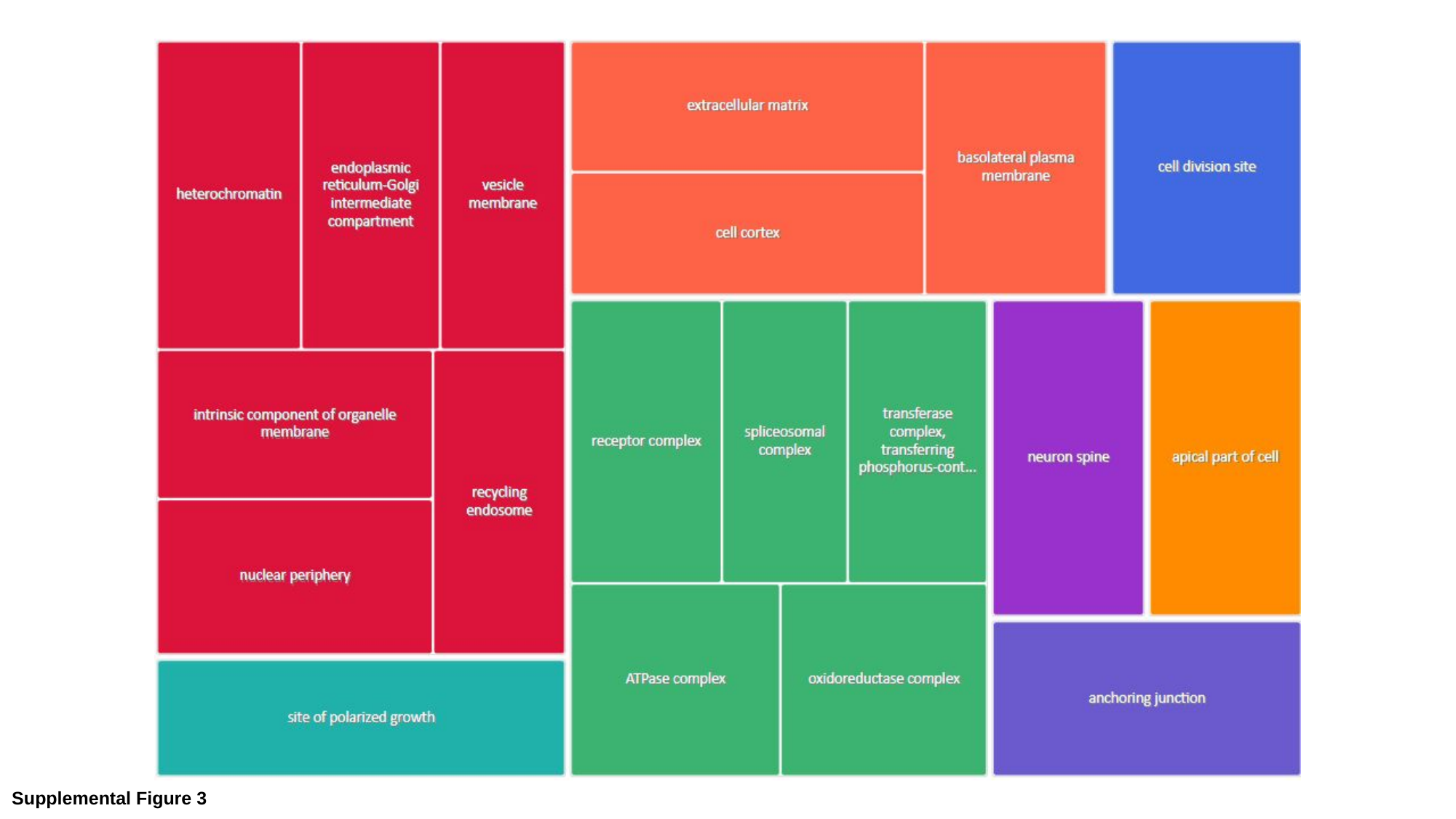

Supplemental Figure 3

Supplement: Supplementary file 22 [file Presentation3.PPTX]

## Slide 1
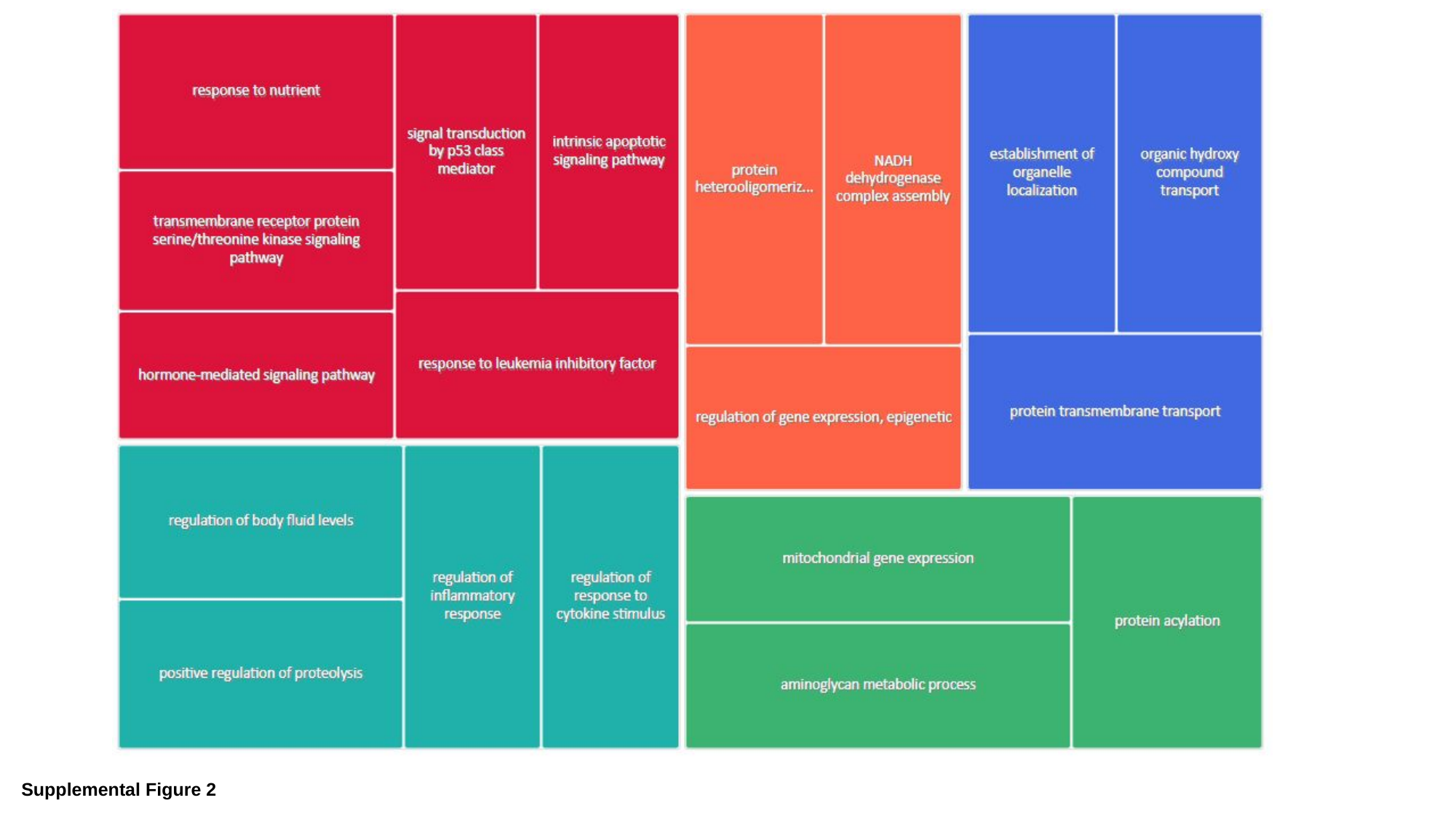

Supplemental Figure 2

Supplement: Supplementary file 24 [file Presentation2.PPTX]

## Slide 1
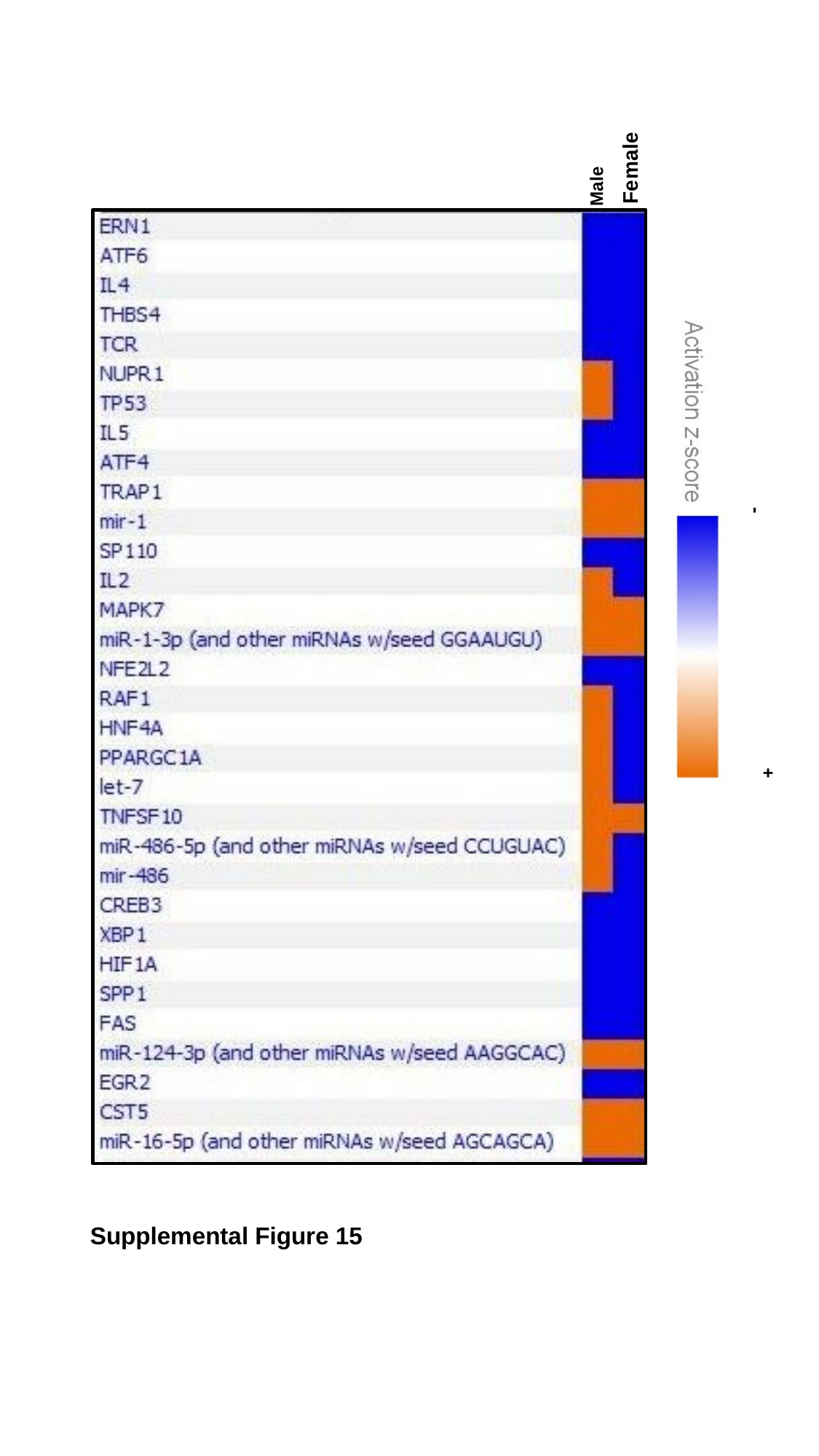

Supplemental Figure 15

Supplement: Supplementary file 25 [file Presentation15.PPTX]

## Slide 1
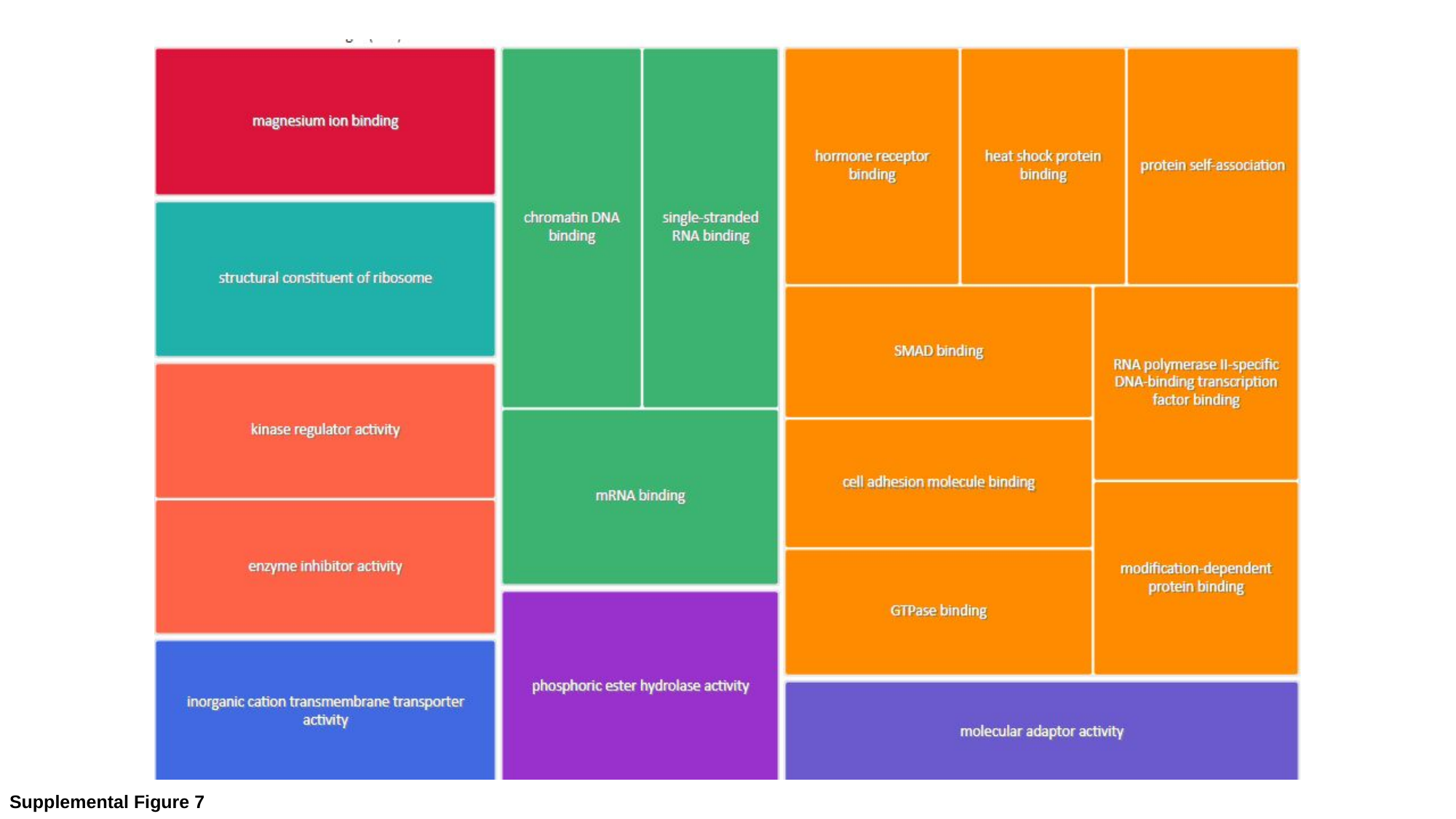

Supplemental Figure 7

Supplement: Supplementary file 28 [file Presentation7.PPTX]

## Slide 1
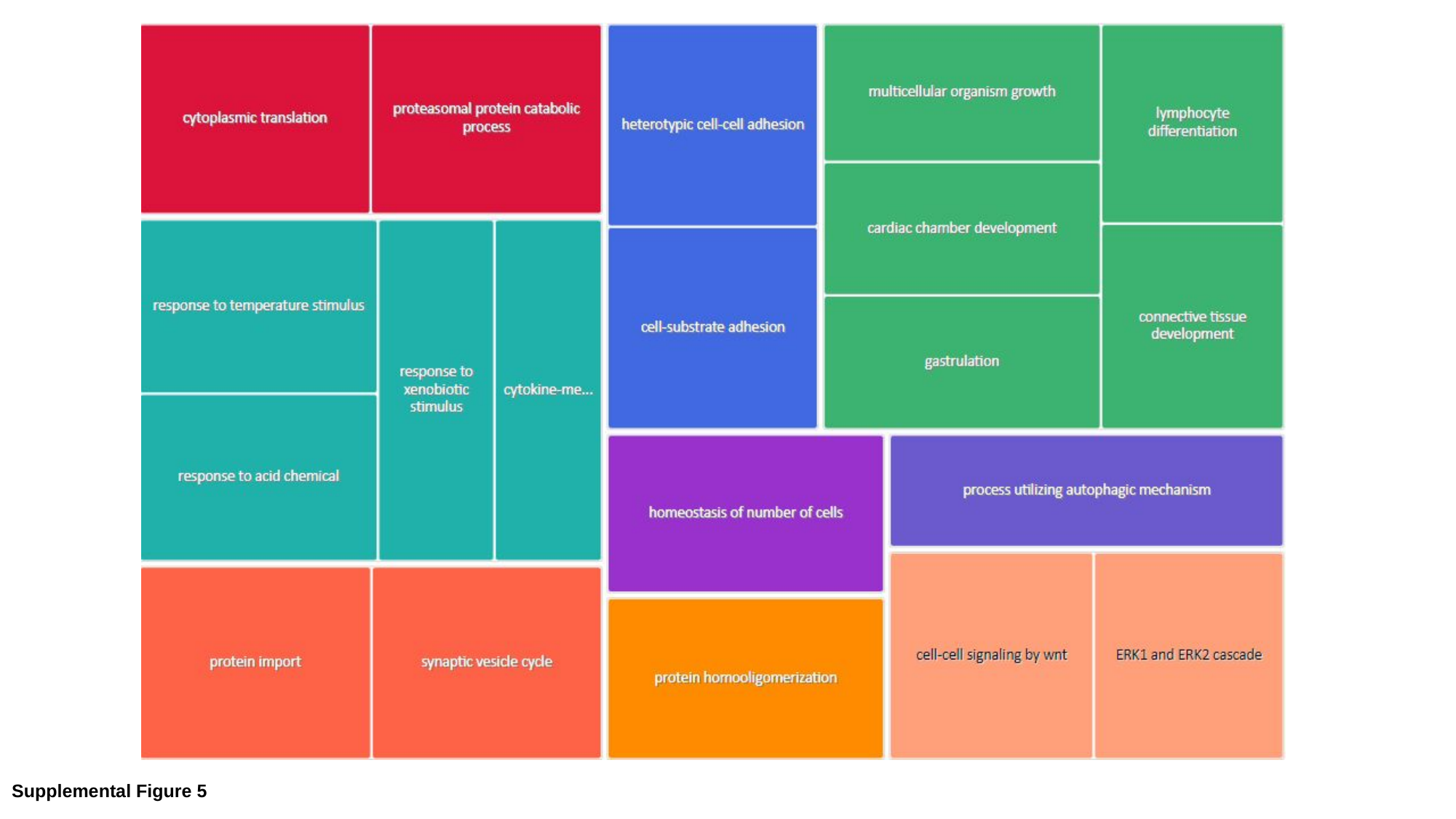

Supplemental Figure 5

Supplement: Supplementary file 31 [file Presentation5.PPTX]
