# Supplementary material for: Maternal obesity alters the placental transcriptome in a fetal sex-dependent manner
Source: Front Cell Dev Biol. 2023 Jun 15;11:1178533. doi: 10.3389/fcell.2023.1178533 (PMC10309565; doi:10.3389/fcell.2023.1178533)
Supplement: Supplementary file 8 [file Table8.DOCX]

**Supplemental Figure 8: KEGG pathway enrichment analysis by GSEA. List of down-regulated KEGG pathways in male placentas of obese dams compared to male placentas of the control group.**

| **Pathway name** | **No of the Genes in the overlap** | **P-value** | **FDRq-value** |
| --- | --- | --- | --- |
| Protein export | 4 | 1.33 e^-4^ | 2.48 e^-2^ |
| DNA replication | 4 | 6.65 e^-4^ | 3.82 e^-2^ |
| One carbon pool by folate | 3 | 8.22 e^-4^ | 3.82 e^-2^ |
| Aminoacyl-tRNA biosynthesis | 4 | 1.09 e^-3^ | 4.07 e^-2^ |
| Purine metabolism | 11 | 2.83 e^-9^ | 5.26 e^-7^ |
| Pyrimidine metabolism | 9 | 6.68 e^-9^ | 6.21 e^-7^ |
| Oxidative phosphorylation | 8 | 1.17 e^-6^ | 5.11 e^-5^ |
| Huntington's disease | 9 | 1.37 e^-6^ | 5.11 e^-5^ |
| Parkinson's disease | 7 | 1.2 e^-5^ | 3.72 e^-4^ |
| RNA polymerase | 4 | 2.38 e^-5^ | 6.33 e^-4^ |
| Ubiquitin mediated proteolysis | 6 | 1.48 e^-4^ | 3.43 e^-3^ |
| Alzheimer's disease | 6 | 4.49 e^-4^ | 8.95 e^-3^ |
| Glycolysis / Gluconeogenesis | 4 | 4.81 e^-4^ | 8.95 e^-3^ |

KEGG, Kyoto Encyclopedia of Genes and Genomes; GSEA, gene set enrichment analysis; FDRq, adjusted q-value.
