# Supplementary material for: Maternal obesity alters the placental transcriptome in a fetal sex-dependent manner
Source: Front Cell Dev Biol. 2023 Jun 15;11:1178533. doi: 10.3389/fcell.2023.1178533 (PMC10309565; doi:10.3389/fcell.2023.1178533)
Supplement: Supplementary file 9 [file Presentation13.PPTX]

## Slide 1
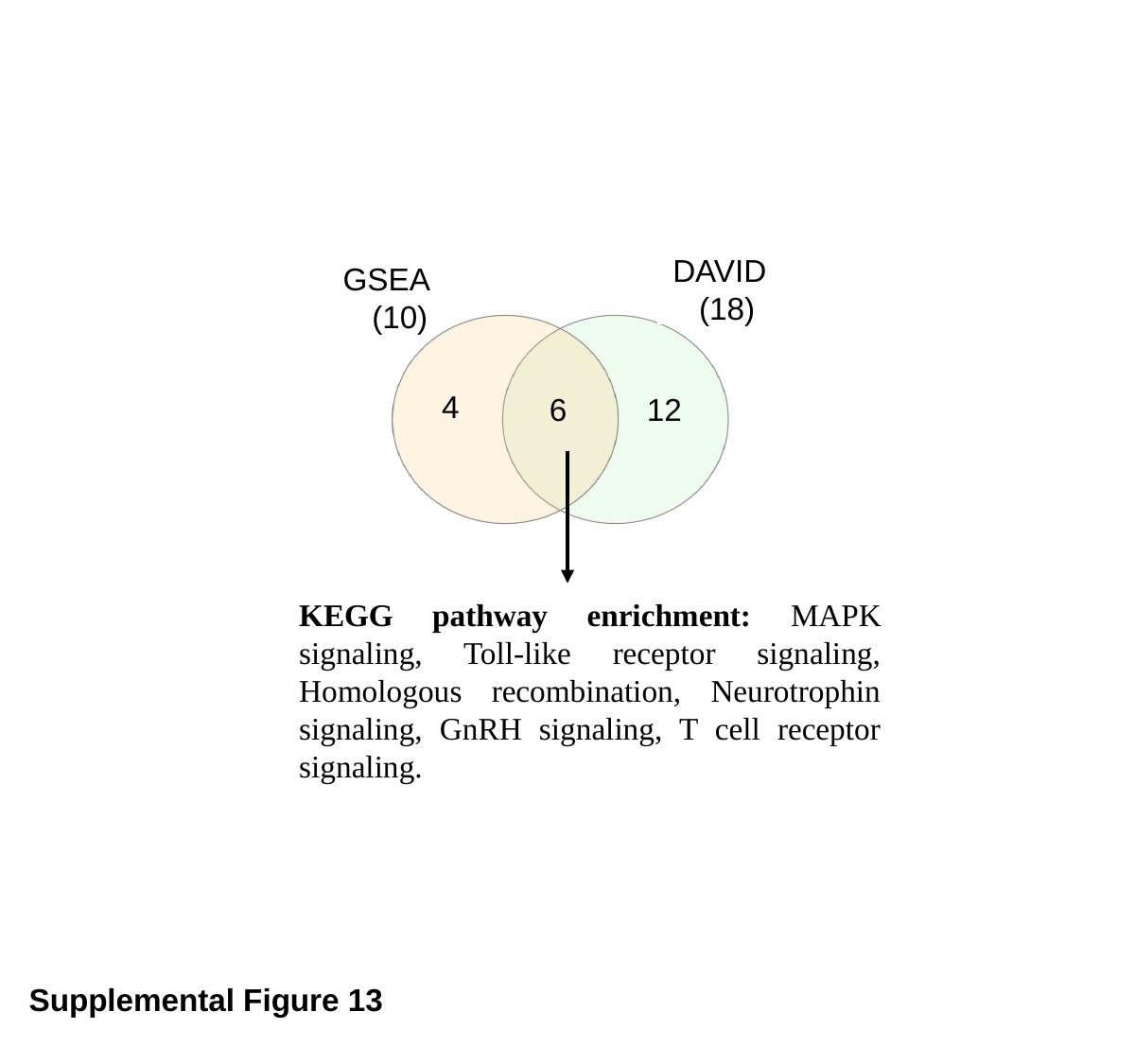

GSEA
 (10)
DAVID
 (18)
4
12
6
KEGG pathway enrichment: MAPK signaling, Toll-like receptor signaling, Homologous recombination, Neurotrophin signaling, GnRH signaling, T cell receptor signaling.
Supplemental Figure 13
