# Supplementary material for: Maternal obesity alters the placental transcriptome in a fetal sex-dependent manner
Source: Front Cell Dev Biol. 2023 Jun 15;11:1178533. doi: 10.3389/fcell.2023.1178533 (PMC10309565; doi:10.3389/fcell.2023.1178533)
Supplement: Supplementary file 10 [file Table12.DOCX]

**Supplemental Table 12: KEGG pathway enrichment analysis by GSEA. List of down-regulated KEGG pathways in female placentas of obese dams compared to the female placentas of the control group.**

| **Pathway name** | **No of the Genes in the overlap** | **P-value** | **FDRq-value** |
| --- | --- | --- | --- |
| Endocytosis | 9 | 1.96 e^-4^ | 2.42 e^-2^ |
| Neurotrophin signaling pathway | 7 | 5.16 e^-4^ | 2.42 e^-2^ |
| Cysteine and methionine metabolism | 4 | 5.2 e^-4^ | 2.42 e^-2^ |
| Fructose and mannose metabolism | 4 | 5.2 e^-4^ | 2.42 e^-2^ |
| Pyrimidine metabolism | 6 | 7.77 e^-4^ | 2.89 e^-2^ |
| Aminoacyl-tRNA biosynthesis | 4 | 1.07 e^-3^ | 3.31 e^-2^ |
| Ubiquitin mediated proteolysis | 6 | 1.62 e-4 | 3 e-2 |

KEGG, Kyoto Encyclopedia of Genes and Genomes; GSEA, gene set enrichment analysis; FDRq, false discovery rate-adjusted q-value.
