# Supplementary material for: Maternal obesity alters the placental transcriptome in a fetal sex-dependent manner
Source: Front Cell Dev Biol. 2023 Jun 15;11:1178533. doi: 10.3389/fcell.2023.1178533 (PMC10309565; doi:10.3389/fcell.2023.1178533)
Supplement: Supplementary file 11 [file Table7.DOCX]

**Supplemental Table 7: KEGG pathway enrichment analysis by DAVID Gene Functional Classification Tool. List of up-regulated KEGG pathway in male obese placentas compared to male control placentas.**

| Pathway name | No of the Genes in the overlap | P-value |
| --- | --- | --- |
| Autophagy - other | 7 | 5.6E-5 |
| Autophagy - animal | 13 | 6.0E-5 |
| Mitophagy - animal | 8 | 6.6E-4 |
| Measles | 10 | 4.6E-3 |
| Inositol phosphate metabolism | 7 | 4.7E-3 |
| Phosphatidylinositol signaling system | 8 | 4.9E-3 |
| Tight junction | 10 | 1.1E-2 |
| Spinocerebellar ataxia | 9 | 1.2E-2 |
| Adipocytokine signaling pathway | 6 | 1.9E-2 |
| T cell receptor signaling pathway | 7 | 2.5E-2 |
| Fatty acid degradation | 5 | 2.6E-2 |
| Hepatitis C | 9 | 2.8E-2 |
| Apoptosis | 8 | 2.9E-2 |
| Apoptosis - multiple species | 4 | 3.2E-2 |
| Alcoholic liver disease | 8 | 3.4E-2 |
| Glycosphingolipid biosynthesis - ganglio series | 3 | 4.1E-2 |
| Leukocyte transendothelial migration | 7 | 4.4E-2 |
| PPAR signaling pathway | 6 | 4.5E-2 |
| Sphingolipid signaling pathway | 7 | 5.4E-2 |
| p53 signaling pathway | 5 | 7.2E-2 |
| Yersinia infection | 7 | 7.4E-2 |
| Wnt signaling pathway | 8 | 8.0E-2 |
| Insulin resistance | 6 | 9.2E-2 |
| NOD-like receptor signaling pathway | 9 | 9.4E-2 |
| Cholesterol metabolism | 4 | 9.5E-2 |
| C-type lectin receptor signaling pathway | 6 | 9.7E-2 |
| Tuberculosis | 8 | 9.8E-2 |
