# Supplementary material for: Maternal obesity alters the placental transcriptome in a fetal sex-dependent manner
Source: Front Cell Dev Biol. 2023 Jun 15;11:1178533. doi: 10.3389/fcell.2023.1178533 (PMC10309565; doi:10.3389/fcell.2023.1178533)
Supplement: Supplementary file 14 [file Table9.DOCX]

**Supplemental Table 9: KEGG pathway enrichment analysis by DAVID Gene Functional Classification Tool. List of down-regulated KEGG pathways in male placentas of obese dams compared to male placentas of the control group.**

| **Pathway name** | **No of the Genes in the overlap** | **P-value** |
| --- | --- | --- |
| Protein processing in the endoplasmic reticulum | 23 | 1.10E-08 |
| Protein export | 9 | 1.70E-06 |
| Parkinson disease | 23 | 1.80E-05 |
| Amyotrophic lateral sclerosis | 24 | 9.10E-04 |
| Prion disease | 19 | 1.40E-03 |
| Ubiquitin mediated proteolysis | 13 | 1.80E-03 |
| Pathways of neurodegeneration - multiple diseases | 26 | 5.00E-03 |
| Purine metabolism | 11 | 7.90E-03 |
| Nucleocytoplasmic transport | 10 | 9.50E-03 |
| Huntington disease | 18 | 1.10E-02 |
| Metabolic pathways | 65 | 1.50E-02 |
| RNA polymerase | 5 | 1.60E-02 |
| Fanconi anemia pathway | 6 | 1.90E-02 |
| DNA replication | 5 | 2.10E-02 |
| Spliceosome | 10 | 2.10E-02 |
| Thyroid hormone synthesis | 7 | 2.50E-02 |
| Alzheimer disease | 20 | 2.50E-02 |
| Carbon metabolism | 9 | 3.20E-02 |
| Biosynthesis of amino acids | 7 | 3.30E-02 |
| Nucleotide excision repair | 5 | 4.10E-02 |
| Nucleotide metabolism | 7 | 4.30E-02 |
| Salmonella infection | 14 | 4.70E-02 |
| Non-alcoholic fatty liver disease | 10 | 5.00E-02 |
| Aminoacyl-tRNA biosynthesis | 6 | 5.10E-02 |
| Thermogenesis | 13 | 5.10E-02 |
| Glycolysis / Gluconeogenesis | 6 | 5.40E-02 |
| Oxidative phosphorylation | 9 | 5.50E-02 |
| Central carbon metabolism in cancer | 6 | 5.90E-02 |
| Rap1 signaling pathway | 12 | 6.40E-02 |
| Spinocerebellar ataxia | 9 | 6.70E-02 |
| Lipid and atherosclerosis | 12 | 6.70E-02 |
| Chemical carcinogenesis - reactive oxygen species | 12 | 7.80E-02 |
| Fluid shear stress and atherosclerosis | 9 | 8.10E-02 |
| Platelet activation | 8 | 8.80E-02 |
| Pyrimidine metabolism | 5 | 9.10E-02 |
| Base excision repair | 4 | 9.6E-2 |
