# Supplementary material for: Maternal obesity alters the placental transcriptome in a fetal sex-dependent manner
Source: Front Cell Dev Biol. 2023 Jun 15;11:1178533. doi: 10.3389/fcell.2023.1178533 (PMC10309565; doi:10.3389/fcell.2023.1178533)
Supplement: Supplementary file 16 [file DataSheet1.PDF]

Supplemental Figure 1: Body weight gain in pregnant mice at different gestational intervals. Data are mean + SEM [n = 5/each group]. Statistical significance was determined using one-way ANOVA followed by Tukey–Kramer multiple comparisons post hoc tests. **$P < 0.05$; ***$P < 0.001$.

Supplemental Figure 2: TreeMap of GO biological processes categories down-regulated in male placentas of obese dams. ROVIGO was used to remove redundant GO terms and to join the cluster representatives (the single rectangles) into superclusters (represented by different colors). Each rectangle's size reflects the GO term's FDR value (larger for lower FDR).

Supplemental Figure 3: TreeMap of GO cellular component categories down-regulated in male placentas of obese dams. ROVIGO was used to remove redundant GO terms and to join the cluster representatives (the single rectangles) into superclusters (represented by different colors). Each rectangle's size reflects the GO term's FDR value (larger for lower FDR).

Supplemental Figure 4: TreeMap of GO molecular function categories down-regulated in male placentas of obese dams. ROVIGO was used to remove redundant GO terms and to join the cluster representatives (the single rectangles) into superclusters (represented by different colors). Each rectangle's size reflects the GO term's FDR value (larger for lower FDR).

Supplemental Figure 5: TreeMap of GO biological processes categories up-regulated in female placentas of obese dams. ROVIGO was used to remove redundant GO terms and to join the cluster representatives (the single rectangles) into superclusters (represented by different colors). Each rectangle's size reflects the GO term's FDR value (larger for lower FDR).

Supplemental Figure 6: TreeMap of GO cellular component categories up-regulated in female placentas of obese dams. ROVIGO was used to remove redundant GO terms and to join the cluster representatives (the single rectangles) into superclusters (represented by different colors). Each rectangle's size reflects the GO term's FDR value (larger for lower FDR).

Supplemental Figure 7: TreeMap of GO molecular function categories up-regulated in female placentas of obese dams. ROVIGO was used to remove redundant GO terms and to join the cluster representatives (the single rectangles) into superclusters (represented by different colors). Each rectangle's size reflects the GO term's FDR value (larger for lower FDR).

Supplemental Figure 8: TreeMap of GO biological processes categories down-regulated in female placentas of obese dams. ROVIGO was used to remove redundant GO terms and to join the cluster representatives (the single rectangles) into superclusters (represented by different colors). Each rectangle's size reflects the GO term's FDR value (larger for lower FDR).

Supplemental Figure 9: TreeMap of GO cellular component categories down-regulated in female placentas of obese dams. ROVIGO was used to remove redundant GO terms and to join the cluster representatives (the single rectangles) into superclusters (represented by different colors). Each rectangle's size reflects the GO term's FDR value (larger for lower FDR).

Supplemental Figure 10: TreeMap of GO molecular function categories down-regulated in female placentas of obese dams. ROVIGO was used to remove redundant GO terms and to join

the cluster representatives (the single rectangles) into superclusters (represented by different colors). Each rectangle's size reflects the GO term's FDR value (larger for lower FDR).

Supplemental Figure 11: Venn diagram illustrating the overlap between the KEGG enrichment analysis results generated using DAVID and GSEA. For the upregulated DEGs in male placentas of obese dams, 10 and 27 KEGG pathways were enriched according to the GSEA and DAVID Functional Annotation tool analysis. KEGG, Kyoto Encyclopedia of Genes and Genomes; DAVID, Database for Annotation Visualization and Integrated Discovery; GSEA, gene set enrichment analysis.

Supplemental Figure 12: Venn diagram illustrating the overlap between the KEGG enrichment analysis results generated using DAVID and GSEA. For the downregulated DEGs in male placentas of obese dams, 13 and 36 KEGG pathways were enriched according to the GSEA and DAVID Functional Annotation tool analysis. KEGG, Kyoto Encyclopedia of Genes and Genomes; DAVID, Database for Annotation Visualization and Integrated Discovery; GSEA, gene set enrichment analysis.

Supplemental Figure 13: Venn diagram illustrating the overlap between the KEGG enrichment analysis results generated using DAVID and GSEA. For the up-regulated DEGs in female placentas of obese dams, 10 and 18 KEGG pathways were enriched according to the GSEA and DAVID Functional Annotation tool analysis. KEGG, Kyoto Encyclopedia of Genes and Genomes; DAVID, Database for Annotation Visualization and Integrated Discovery; GSEA, gene set enrichment analysis.

Supplemental Figure 14: Venn diagram illustrating the overlap between the KEGG enrichment analysis results generated using DAVID and GSEA. For the downregulated DEGs in female placentas of obese dams, 7 and 16 KEGG pathways were enriched according to the GSEA and DAVID Functional Annotation tool analysis. KEGG, Kyoto Encyclopedia of Genes and Genomes; DAVID, Database for Annotation Visualization and Integrated Discovery; GSEA, gene set enrichment analysis.

Supplemental Figure 15: Maternal obesity alters placental upstream regulators in a fetal sex-dependent manner.
