# Supplementary material for: Maternal obesity alters the placental transcriptome in a fetal sex-dependent manner
Source: Front Cell Dev Biol. 2023 Jun 15;11:1178533. doi: 10.3389/fcell.2023.1178533 (PMC10309565; doi:10.3389/fcell.2023.1178533)
Supplement: Supplementary file 17 [file Table5.DOCX]

| **Gene Name** | **Gene ID** | **Male** | | **Female** | |
| --- | --- | --- | --- | --- | --- |
|  |  | **P-Value** | **Fold change** | **P-Value** | **Fold change** |
| Myosin light chain 1/3, skeletal muscle isoform | Myl1 | 0.032 | 1.26E+4 | 0.028 | 1.35E+4 |
| Expressed sequence AU022751 | AU022751 | 0.006 | 9.05E+2 | 0.022 | 9.50 |
| Heat shock factor protein 1 | Hsf1 | 0.019 | 5.73 | 0.038 | 1.65 |
| Ribosyldihydronicotinamide dehydrogenase [quinone] | Nqo2 | 0.028 | 5.46 | 0.051 | 5.46 |
| Dynein light chain Tctex-type 1F | Dynlt1f | 0.027 | 2.80 | 0.046 | 2.55 |
| Probable G-protein coupled receptor 160 | Gpr160 | 0.036 | 4.42 | 0.046 | 1.57 |
| Leucine-rich repeat-containing protein 2 | Lrrc2 | 0.019 | 4.16 | 0.050 | 1.85 |
| Ribosome-releasing factor 2, mitochondrial | Gfm2 | 0.035 | 4.11 | 0.035 | 5.55 |
| Protein yippee-like 5 | Ypel5 | 0.009 | 3.89 | 0.022 | 1.24 |
| PQ-loop repeat-containing protein 1 | Pqlc1 | 0.038 | 3.70 | 0.019 | 3.72 |
| Beta-adducin | Add2 | 0.016 | 3.42 | 0.043 | 2.61 |
| Zinc finger protein 219 | Zfp219 | 0.007 | 3.24 | 0.051 | 1.96 |
| Ubiquitin carboxyl-terminal hydrolase MINDY-3 | Mindy3 | 0.016 | 1.62 | 0.005 | 4.14 |
| Kinesin-like protein KIF21A | Kif21a | 0.045 | 3.10 | 0.053 | 5.59 |
| 1600012H06Rik | 1600012H06Rik | 0.050 | 2.80 | 0.050 | 2.42 |
| Toll-like receptor 7 | Tlr7 | 0.044 | 2.59 | 0.050 | 4.53 |
| Signal-induced proliferation-associated protein 1 | Sipa1 | 0.045 | 2.16 | 0.012 | 11.50 |
| Vezatin | Vezt | 0.028 | 2.05 | 0.023 | 1.63 |
| DNA replication licensing factor MCM6 | Mcm6 | 0.017 | 2.00 | 0.023 | 1.96 |
| Mesoderm induction early response protein 1 | Mier1 | 0.023 | 1.98 | 0.053 | 1.97 |
| HMG box-containing protein 1 | Hbp1 | 0.033 | 1.94 | 0.026 | 1.15 |
| Autophagy-related protein 13 | Atg13 | 0.053 | 1.61 | 0.045 | 1.70 |
| Nebulin | Neb | 0.034 | 1.88 | 0.045 | 1.83 |
| Transmembrane protein 178A | Tmem178 | 0.031 | 1.86 | 0.008 | 2.52 |
| Choline/ethanolamine kinase | Chkb | 0.056 | 1.73 | 0.041 | 4.23 |
| Mitofusin-2 | Mfn2 | 0.052 | 1.72 | 0.018 | 1.80 |
| Transcription activator BRG1 | Smarca4 | 0.048 | 1.71 | 0.050 | 1.77 |
| G-protein-signaling modulator 1 | Gpsm1 | 0.049 | 1.69 | 0.011 | 2.10 |
| Ras-related protein Rab-43 | Rab43 | 0.033 | 1.51 | 0.038 | 1.50 |

**Supplemental Table 5:** List of differentially expressed genes (DEGs) upregulated in male and female placenta (obese vs. control group).

| **Gene Name** | **Gene ID** | **Male** | | **Female** | |
| --- | --- | --- | --- | --- | --- |
|  |  | **P-Value** | **Fold change** | **P-Value** | **Fold change** |
| Protein lin-54 homolog | Lin54 | 0.054 | 1.67 | 0.013 | 2.09 |
| ETS-related transcription factor Elf-1 | Elf1 | 0.022 | 1.67 | 0.037 | 1.27 |
| Transmembrane protein 209 | Tmem209 | 0.038 | 1.64 | 0.004 | 2.62 |
| CD99 antigen-like protein 2 | Cd99l2 | 0.035 | 1.64 | 0.044 | 1.58 |
| Tensin-2 | Tns2 | 0.002 | 1.62 | 0.048 | 1.32 |
| Mitochondrial glutamate carrier 1 | Slc25a22 | 0.030 | 1.62 | 0.026 | 1.71 |
| DNA endonuclease RBBP8 | Rbbp8 | 0.009 | 1.59 | 0.005 | 2.54 |
| Sentrin-specific protease 2 | Senp2 | 0.050 | 1.59 | 0.019 | 1.81 |
| Reticulophagy regulator 1 | Retreg1 | 0.033 | 1.58 | 0.020 | 1.66 |
| Charged multivesicular body protein 4c | Chmp4c | 0.029 | 1.58 | 0.054 | 1.62 |
| TSC22 domain family protein 3 | Tsc22d3 | 0.051 | 1.56 | 0.050 | 2.02 |
| CD2 antigen cytoplasmic tail-binding protein | Cd2bp2 | 0.050 | 2.02 | 0.028 | 3.05 |
| Netrin-4 | Ntn4 | 0.013 | 1.55 | 0.027 | 1.44 |
| RNA-binding protein EWS | Ewsr1 | 0.047 | 1.53 | 0.027 | 1.64 |
| Zinc finger protein 287 | Zfp287 | 0.038 | 1.53 | 0.045 | 1.51 |
| Tumor protein p53-inducible nuclear protein 1 | Trp53inp1 | 0.018 | 1.50 | 0.015 | 1.54 |
| Phosphodiesterase | Pde4b | 0.050 | 1.48 | 0.006 | 1.84 |
| NEDD4-binding protein 2-like 1 | N4bp2l1 | 0.012 | 1.45 | 0.024 | 1.38 |
| Zinc finger protein 22 | Zfp422 | 0.042 | 1.40 | 0.017 | 1.51 |
| Lysine-specific histone demethylase 1A | Kdm1a | 0.012 | 1.40 | 0.050 | 1.28 |
| HMG box transcription factor BBX | Bbx | 0.019 | 1.35 | 0.023 | 1.52 |
| Mitogen-activated protein kinase | Map3k8 | 0.038 | 1.34 | 0.036 | 1.34 |
| Protein Tob1 | Tob1 | 0.013 | 1.34 | 0.044 | 1.26 |
| Transmembrane protein 43 | Tmem43 | 0.050 | 1.34 | 0.046 | 1.40 |
| Glutaredoxin-2, mitochondrial | Glrx2 | 0.048 | 1.33 | 0.023 | 5.77 |
| DNA-binding death effector domain-containing protein 2 | Dedd2 | 0.026 | 1.33 | 0.048 | 1.26 |
| Dual specificity protein phosphatase 10 | Dusp10 | 0.048 | 1.33 | 0.017 | 1.43 |

| **Gene Name** | **Gene ID** | **Male** | | **Female** | |
| --- | --- | --- | --- | --- | --- |
|  |  | **P-Value** | **Fold change** | **P-Value** | **Fold change** |
| Plasminogen activator inhibitor 1 RNA-binding protein | Serbp1 | 0.050 | 1.30 | 0.028 | 1.33 |
| V-set and immunoglobulin domain-containing protein 10 | Vsig10 | 0.031 | 1.29 | 0.007 | 1.44 |
| Terminal uridylyl transferase 7 | Zcchc6 | 0.014 | 1.23 | 0.037 | 1.19 |
| Melanoma-associated antigen F1 | Magef1 | 0.033 | 1.22 | 0.018 | 1.25 |
| DnaJ homolog subfamily B member 6 | Dnajb6 | 0.048 | 1.20 | 0.010 | 2.99 |
| Mitogen-activated protein kinase 6 | Map3k6 | 0.050 | 1.19 | 0.011 | 1.28 |
| Protein FAM102A | Fam102a | 0.037 | 1.17 | 0.023 | 1.17 |
| Ras-related protein Rab-9 | Rab9 | 0.050 | 1.15 | 0.049 | 1.14 |
| Cyclin-L2 | Ccnl2 | 0.043 | 1.12 | 0.008 | 1.17 |
