# Supplementary material for: Maternal obesity alters the placental transcriptome in a fetal sex-dependent manner
Source: Front Cell Dev Biol. 2023 Jun 15;11:1178533. doi: 10.3389/fcell.2023.1178533 (PMC10309565; doi:10.3389/fcell.2023.1178533)
Supplement: Supplementary file 18 [file Table13.DOCX]

**Supplemental Table 13: KEGG pathway enrichment analysis by DAVID Gene Functional Classification Tool. List of down-regulated KEGG pathways in female placentas of obese dams compared to the female placentas of the control group.**

| **Pathway name** | **No of the Genes in the overlap** | **P-value** |
| --- | --- | --- |
| Protein processing in the endoplasmic reticulum | 21 | 4.60E-03 |
| Glycerophospholipid metabolism | 66 | 8.30E-03 |
| Metabolic pathways | 9 | 1.20E-02 |
| Ubiquitin mediated proteolysis | 11 | 1.60E-02 |
| N-Glycan biosynthesis | 6 | 2.30E-02 |
| Pantothenate and CoA biosynthesis | 4 | 3.10E-02 |
| Endocytosis | 15 | 4.20E-02 |
| Axon guidance | 11 | 5.30E-02 |
| Central carbon metabolism in cancer | 6 | 5.70E-02 |
| Endocrine resistance | 7 | 5.80E-02 |
| Nucleocytoplasmic transport | 8 | 6.10E-02 |
| Non-small cell lung cancer | 6 | 7.50E-02 |
| Cysteine and methionine metabolism | 5 | 8.10E-02 |
| Biosynthesis of cofactors | 9 | 8.30E-02 |
| Pyrimidine metabolism | 5 | 9.20E-02 |
| Parkinson disease | 13 | 4.60E-03 |
