# Supplementary material for: Maternal obesity alters the placental transcriptome in a fetal sex-dependent manner
Source: Front Cell Dev Biol. 2023 Jun 15;11:1178533. doi: 10.3389/fcell.2023.1178533 (PMC10309565; doi:10.3389/fcell.2023.1178533)
Supplement: Supplementary file 21 [file Table4.DOCX]

**Supplemental Table 4:** List of differentially expressed genes (DEGs) downregulated in both male and female placenta (Obese vs control diet group).

| **Gene Name** | **Gene ID** | **Male** | | **Female** | |
| --- | --- | --- | --- | --- | --- |
|  |  | **P-Value** | **Fold change** | **P-Value** | **Fold change** |
| Zinc finger and BTB domain-containing protein 7B | Zbtb7b | 0.007 | -2.83 | 0.038 | -2.26 |
| Zinc finger and SCAN domain-containing 22 | Zscan22 | 0.023 | -3.08 | 0.002 | -1.5 |
| Required for meiotic nuclear division protein 1 homolog | Rmnd1 | 0.030 | -5.33 | 0.035 | -2.61 |
| A730036I17Rik | A730036I17Rik | 0.005 | -4.97 | 0.050 | -2.97 |
| Tyrosine-protein kinase Fyn | Fyn | 0.026 | -4.90 | 0.012 | -1.70 |
| Activity-dependent neuroprotector homeobox protein | Adnp | 0.053 | -4.32 | 0.021 | -1.51 |
| Uncharacterized aarF domain-containing protein kinase | Adck1 | 0.020 | -3.85 | 0.044 | -2.27 |
| Guanine nucleotide exchange factor DBS | Mcf2l | 0.047 | -3.55 | 0.038 | -1.47 |
| Beta-1,4-galactosyltransferase 4 | B4galt4 | 0.001 | -3.17 | 0.018 | -1.90 |
| Myosin light chain 4 | Myl4 | 0.012 | -3.01 | 0.043 | -2.13 |
| Phospholipase B1, membrane-associated | Plb1 | 0.001 | -2.94 | 0.047 | -1.74 |
| E3 ubiquitin-protein ligase rififylin | Rffl | 0.016 | -2.89 | 0.016 | -1.53 |
| Glutamate receptor ionotropic, kainate 3 | Grik3 | 0.044 | -2.61 | 0.012 | -2.87 |
| DET1- and DDB1-associated protein 1 | Dda1 | 0.018 | -2.46 | 0.042 | -2.51 |
| TOX high mobility group box family member 2 | Tox2 | 0.006 | -2.38 | 0.032 | -1.81 |
| DNA replication ATP-dependent helicase/nuclease DNA2 | Dna2 | 0.045 | -1.60 | 0.025 | -1.96 |
| Calcium/calmodulin-dependent protein kinase type II subunit delta | Camk2d | 0.037 | -2.25 | 0.049 | -1.56 |
| Zinc finger protein ZFAT | Zfat | 0.040 | -2.19 | 0.056 | -1.83 |
| Probable ATP-dependent RNA helicase DDX17 | Ddx17 | 0.019 | -2.14 | 0.041 | -1.30 |
| Xylulose kinase | Xylb | 0.012 | -2.10 | 0.046 | -1.80 |
| Prolactin-2B1 | Prl2b1 | 0.006 | -2.04 | 0.008 | -1.97 |
| CASP8 and FADD-like apoptosis regulator | Cflar | 0.032 | -2.02 | 0.029 | -2.02 |
| C-Jun-amino-terminal kinase-interacting protein 3 | Mapk8ip3 | 0.050 | -1.99 | 0.013 | -2.63 |
| Zfp296 | Zfp296 | 0.051 | -1.93 | 0.017 | -2.23 |
| Cyclin-dependent kinase-like | Cdkl4 | 0.035 | -1.92 | 0.044 | -1.82 |
| Protein LDOC1 | Ldoc1 | 0.023 | -1.92 | 0.054 | -1.56 |
| mRNA turnover protein 4 homolog | Mrto4 | 0.008 | -1.90 | 0.027 | -2.36 |

| **Gene Name** | **Gene ID** | **Male** | | **Female** | |
| --- | --- | --- | --- | --- | --- |
|  |  | **P-Value** | **Fold change** | **P-Value** | **Fold change** |
| Zinc finger, MYND-type-containing 8 | Zmynd8 | 0.009 | -1.88 | 0.054 | -1.64 |
| F-box/WD repeat-containing protein 11 | Fbxw11 | 0.021 | -1.78 | 0.024 | -1.68 |
| Zinc finger protein 131 | Zfp131 | 0.047 | -1.73 | 0.006 | -6.18 |
| Phenylalanine--tRNA ligase beta subunit | Farsb | 0.029 | -1.70 | 0.052 | -1.56 |
| Vacuolar protein sorting-associated protein 8 homolog | Vps8 | 0.011 | -1.63 | 0.018 | -1.52 |
| Zinc finger protein 493 | Zfp493 | 0.031 | -1.57 | 0.047 | -1.52 |
| Adhesion G protein-coupled receptor A3 | Adgra3 | 0.026 | -1.57 | 0.044 | -1.44 |
| Somatoliberin | Ghrh | 0.004 | -1.54 | 0.006 | -1.55 |
| Proline-serine-threonine phosphatase-interacting protein 1 | Pstpip1 | 0.022 | -1.53 | 0.026 | -1.44 |
| SH3 and PX domain-containing protein 2A | Sh3pxd2a | 0.009 | -1.52 | 0.036 | -1.41 |
| Protein disulfide-isomerase A4 | Pdia4 | 0.038 | -1.51 | 0.043 | -1.51 |
| Serine hydroxymethyltransferase, mitochondrial | Shmt2 | 0.002 | -1.51 | 0.003 | -2.68 |
| Endoplasmic reticulum chaperone BiP | Hspa5 | 0.028 | -1.50 | 0.035 | -1.49 |
| AN1-type zinc finger protein 4 | Zfand4 | 0.030 | -1.48 | 0.045 | -3.95 |
| Tyrosyl-DNA phosphodiesterase 1 | Tdp1 | 0.022 | -1.47 | 0.016 | -1.49 |
| Serpin H1 | Serpinh1 | 0.023 | -1.47 | 0.022 | -1.50 |
| Endoplasmin | Hsp90b1 | 0.044 | -1.47 | 0.044 | -1.49 |
| Arylsulfatase B | Arsb | 0.009 | -1.46 | 0.030 | -1.38 |
| Hypoxia up-regulated protein 1 | Hyou1 | 0.041 | -1.44 | 0.022 | -1.53 |
| Mesencephalic astrocyte-derived neurotrophic factor | Manf | 0.050 | -1.43 | 0.044 | -1.48 |
| Vascular endothelial growth factor A | Vegfa | 0.050 | -1.42 | 0.024 | -1.57 |
| Hexokinase-2 | Hk2 | 0.005 | -1.42 | 0.027 | -1.28 |
| 1600014C10Rik | 1600014C10Rik | 0.042 | -1.40 | 0.021 | -1.39 |
| Glycerol-3-phosphate dehydrogenase, mitochondrial | Gpd2 | 0.045 | -1.40 | 0.012 | -1.53 |
| Heat shock protein 75 kDa, mitochondrial | Trap1 | 0.028 | -1.38 | 0.050 | -1.31 |
| EEF1A lysine methyltransferase 4 | Eef1akmt4 | 0.021 | -1.38 | 0.016 | -1.47 |
| Metalloendopeptidase OMA1, mitochondrial | Oma1 | 0.020 | -1.38 | 0.032 | -1.28 |
| Stromal cell-derived factor 1 | Cxcl12 | 0.010 | -1.37 | 0.052 | -1.47 |

| **Gene Name** | **Gene ID** | **Male** | | **Female** | |
| --- | --- | --- | --- | --- | --- |
|  |  | **P-Value** | **Fold change** | **P-Value** | **Fold change** |
| SWI/SNF-related matrix-associated actin-dependent regulator of chromatin subfamily A containing DEAD/H box 1 | Smarcad1 | 0.050 | -1.34 | 0.050 | -1.78 |
| Echinoderm microtubule-associated protein-like 4 | Eml4 | 0.001 | -1.34 | 0.053 | -2.25 |
| Chitinase domain-containing protein 1 | Chid1 | 0.015 | -1.34 | 0.020 | -1.32 |
| Structural maintenance of chromosomes flexible hinge domain-containing protein 1 | Smchd1 | 0.012 | -1.33 | 0.033 | -1.24 |
| STE20/SPS1-related proline-alanine-rich protein kinase | Stk39 | 0.049 | -1.33 | 0.059 | -1.30 |
| Vacuolar protein sorting-associated protein 13A | Vps13a | 0.008 | -1.32 | 0.005 | -1.34 |
| Serine/threonine-protein kinase 36 | Stk36 | 0.045 | -1.32 | 0.051 | -1.30 |
| 2310057M21Rik | 2310057M21Rik | 0.018 | -1.31 | 0.023 | -1.29 |
| DENN domain-containing protein 4C | Dennd4c | 0.042 | -1.31 | 0.038 | -1.28 |
| Protein GPR108 | Gpr108 | 0.016 | -3.51 | 0.050 | -1.86 |
| Coiled-coil domain-containing protein 107 | Ccdc107 | 0.020 | -1.30 | 0.004 | -1.40 |
| Thyroid adenoma-associated protein homolog | Thada | 0.045 | -1.28 | 0.014 | -1.37 |
| DnaJ homolog subfamily C member 3 | Dnajc3 | 0.044 | -1.28 | 0.026 | -1.33 |
| Nodal modulator 1 | Nomo1 | 0.013 | -1.27 | 0.005 | -1.34 |
| Leucine--tRNA ligase, cytoplasmic | Lars | 0.009 | -1.27 | 0.017 | -1.24 |
| Staphylococcal nuclease domain-containing protein 1 | Snd1 | 0.001 | -1.27 | 0.012 | -1.19 |
| RUS1 family protein C16orf58 homolog | BC017158 | 0.013 | -1.27 | 0.015 | -1.26 |
| Obscurin-like protein 1 | Obsl1 | 0.025 | -1.27 | 0.041 | -1.23 |
| Oligosaccharyltransferase complex subunit OSTC | Ostc | 0.011 | -1.26 | 0.032 | -1.22 |
| 28S ribosomal protein S35, mitochondrial | Mrps35 | 0.003 | -1.25 | 0.050 | -1.14 |
| Calnexin | Canx | 0.013 | -1.25 | 0.014 | -1.24 |
| MICOS complex subunit Mic19 | Chchd3 | 0.015 | -1.25 | 0.017 | -1.22 |
| Mitochondrial import receptor subunit TOM5 homolog | Tomm5 | 0.022 | -1.24 | 0.011 | -1.26 |

| **Gene Name** | **Gene ID** | **Male** | | **Female** | |
| --- | --- | --- | --- | --- | --- |
|  |  | **P-Value** | **Fold change** | **P-Value** | **Fold change** |
| NADH dehydrogenase [ubiquinone] 1 alpha subcomplex subunit 10, mitochondrial | Ndufa10 | 0.005 | -1.23 | 0.032 | -1.16 |
| Nuclear transport factor 2 | Nutf2 | 0.029 | -1.23 | 0.045 | -1.96 |
| CDC42 small effector protein 2 | Cdc42se2 | 0.044 | -1.22 | 0.032 | -1.25 |
| Dolichyl-diphosphooligosaccharide--protein glycosyltransferase subunit 1 | Rpn1 | 0.006 | -1.22 | 0.009 | -1.21 |
| Glycosylphosphatidylinositol anchor attachment 1 protein | Gpaa1 | 0.017 | -1.21 | 0.009 | -1.25 |
| DNA-directed RNA polymerases I, II, and III subunit RPABC | Polr2h | 0.020 | -1.21 | 0.041 | -1.18 |
| Signal recognition particle receptor subunit beta | Srprb | 0.022 | -1.21 | 0.050 | -1.18 |
| Palmitoyltransferase ZDHHC16 | Zdhhc16 | 0.047 | -1.21 | 0.041 | -1.21 |
| Nectin-2 | Nectin2 | 0.045 | -1.20 | 0.019 | -1.24 |
| Phosphoglycerate mutase 1 | Pgam1 | 0.004 | -1.20 | 0.027 | -1.14 |
| 39S ribosomal protein L37, mitochondria | Mrpl37 | 0.025 | -1.20 | 0.022 | -1.21 |
| Dolichyl-diphosphooligosaccharide--protein glycosyltransferase subunit STT3 | Stt3b | 0.045 | -1.20 | 0.054 | -1.18 |
| Glyoxalase domain-containing protein 4 | Glod4 | 0.002 | -1.19 | 0.003 | -1.18 |
| Cytochrome P450 CYP4F13 | Cyp4f13 | 0.044 | -1.19 | 0.024 | -1.23 |
| Prostaglandin E synthase 2 | Gbf1 | 0.024 | -1.19 | 0.039 | -1.17 |
| Kinesin-like protein KIF2A | Kif2a | 0.038 | -1.18 | 0.031 | -1.19 |
| E3 ubiquitin-protein ligase HERC2 | Herc2 | 0.008 | -1.16 | 0.004 | -1.19 |
| Nuclear receptor coactivator 5 | Ncoa5 | 0.007 | -1.16 | 0.006 | -1.17 |
| Protein kinase C iota typ | Prkci | 0.048 | -1.16 | 0.017 | -1.20 |
| Heterogeneous nuclear ribonucleoprotein U | Hnrnpu | 0.007 | -1.16 | 0.053 | -1.10 |
| Vigilin | Hdlbp | 0.043 | -1.16 | 0.031 | -1.28 |
| Rab-like protein 6 | Rabl6 | 0.026 | -1.15 | 0.003 | -1.23 |
| Tripartite motif-containing protein 44 | Trim44 | 0.001 | -1.15 | 0.034 | -1.07 |
| NAD kinase | Nadk | 0.057 | -1.15 | 0.022 | -1.19 |
| Importin subunit alpha-5 | Kpna1 | 0.017 | -1.15 | 0.003 | -1.19 |
| Sec24-related gene family, member B (S. cerevisiae) | Sec24b | 0.022 | -1.12 | 0.024 | -1.12 |
