# Supplementary material for: Maternal obesity alters the placental transcriptome in a fetal sex-dependent manner
Source: Front Cell Dev Biol. 2023 Jun 15;11:1178533. doi: 10.3389/fcell.2023.1178533 (PMC10309565; doi:10.3389/fcell.2023.1178533)
Supplement: Supplementary file 23 [file Presentation12.PPTX]

## Slide 1
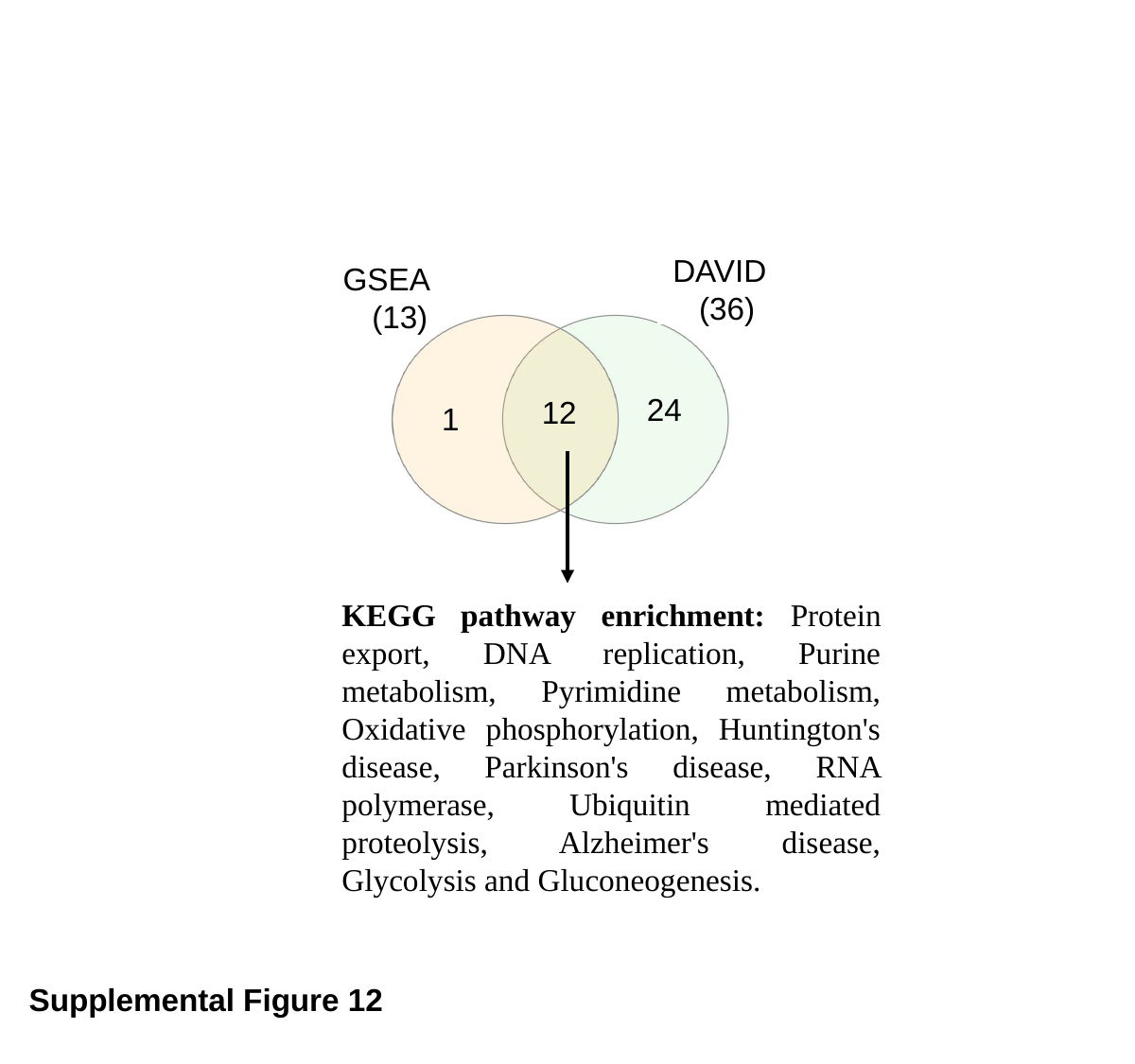

GSEA
 (13)
DAVID
 (36)
24
12
1
KEGG pathway enrichment: Protein export, DNA replication, Purine metabolism, Pyrimidine metabolism, Oxidative phosphorylation, Huntington's disease, Parkinson's disease, RNA polymerase, Ubiquitin mediated proteolysis, Alzheimer's disease, Glycolysis and Gluconeogenesis.
Supplemental Figure 12
