# Supplementary material for: Maternal obesity alters the placental transcriptome in a fetal sex-dependent manner
Source: Front Cell Dev Biol. 2023 Jun 15;11:1178533. doi: 10.3389/fcell.2023.1178533 (PMC10309565; doi:10.3389/fcell.2023.1178533)
Supplement: Supplementary file 26 [file Table11.DOCX]

**Supplemental Table 11: KEGG pathway enrichment analysis by DAVID Gene Functional Classification Tool. List of upregulated KEGG pathways in female placentas of obese dams compared to the female placentas of the control group.**

| **Pathway name** | **No of the Genes in the overlap** | **P-value** |
| --- | --- | --- |
| MAPK signaling pathway | 18 | 5.60E-02 |
| TNF signaling pathway | 8 | 1.20E-02 |
| Homologous recombination | 5 | 1.20E-02 |
| Toll-like receptor signaling pathway | 7 | 2.20E-02 |
| Thermogenesis | 11 | 3.00E-02 |
| GnRH signaling pathway | 6 | 4.60E-02 |
| Longevity regulating pathway | 6 | 4.60E-02 |
| Cytosolic DNA-sensing pathway | 5 | 4.80E-02 |
| Adrenergic signaling in cardiomyocytes | 8 | 4.80E-02 |
| Neurotrophin signaling pathway | 7 | 4.90E-02 |
| Hepatitis B | 8 | 6.50E-02 |
| T cell receptor signaling pathway | 6 | 7.40E-02 |
| Spinocerebellar ataxia | 7 | 8.70E-02 |
| Alcoholic liver disease | 7 | 8.70E-02 |
| Autophagy - animal | 7 | 9.00E-02 |
| Rap1 signaling pathway | 9 | 9.60E-02 |
| Measles | 7 | 9.90E-02 |
| Autophagy - animal | 9 | 5.60E-02 |
