# Supplementary material for: Maternal obesity alters the placental transcriptome in a fetal sex-dependent manner
Source: Front Cell Dev Biol. 2023 Jun 15;11:1178533. doi: 10.3389/fcell.2023.1178533 (PMC10309565; doi:10.3389/fcell.2023.1178533)
Supplement: Supplementary file 27 [file Table6.DOCX]

| Pathway name | No of the Genes in overlap | P-value | FDRq-value |
| --- | --- | --- | --- |
| Tight junction | 9 | 1.23 e^-5^ | 2.12 e^-3^ |
| Inositol phosphate metabolism | 6 | 2.28 e^-5^ | 2.12 e^-3^ |
| Adipocytokine signaling pathway | 6 | 7.85 e^-5^ | 3.44 e^-3^ |
| Fatty acid metabolism | 5 | 7.99 e^-5^ | 3.44 e^-3^ |
| PPAR signaling pathway | 6 | 9.26 e^-5^ | 3.44 e^-3^ |
| Phosphatidylinositol signaling system | 6 | 1.59 e^-4^ | 4.92 e^-3^ |
| Glycosphingolipid biosynthesis - ganglio series | 3 | 4.87 e^-4^ | 1.29 e^-2^ |
| Endocytosis | 8 | 7.34 e^-4^ | 1.71 e^-2^ |
| T cell receptor signaling pathway | 6 | 1.05 e^-3^ | 2.16 e^-2^ |
| Wnt signaling pathway | 7 | 1.18 e^-3^ | 2.2 e^-2^ |

**Supplemental Table 6: KEGG pathway enrichment analysis by GSEA. List of up-regulated KEGG pathway in male obese placentas compared to male control placentas.**

KEGG, Kyoto Encyclopedia of Genes and Genomes; GSEA, gene set enrichment analysis; FDRq, adjusted q-value.
