# Supplementary material for: Maternal obesity alters the placental transcriptome in a fetal sex-dependent manner
Source: Front Cell Dev Biol. 2023 Jun 15;11:1178533. doi: 10.3389/fcell.2023.1178533 (PMC10309565; doi:10.3389/fcell.2023.1178533)
Supplement: Supplementary file 29 [file Presentation11.PPTX]

## Slide 1
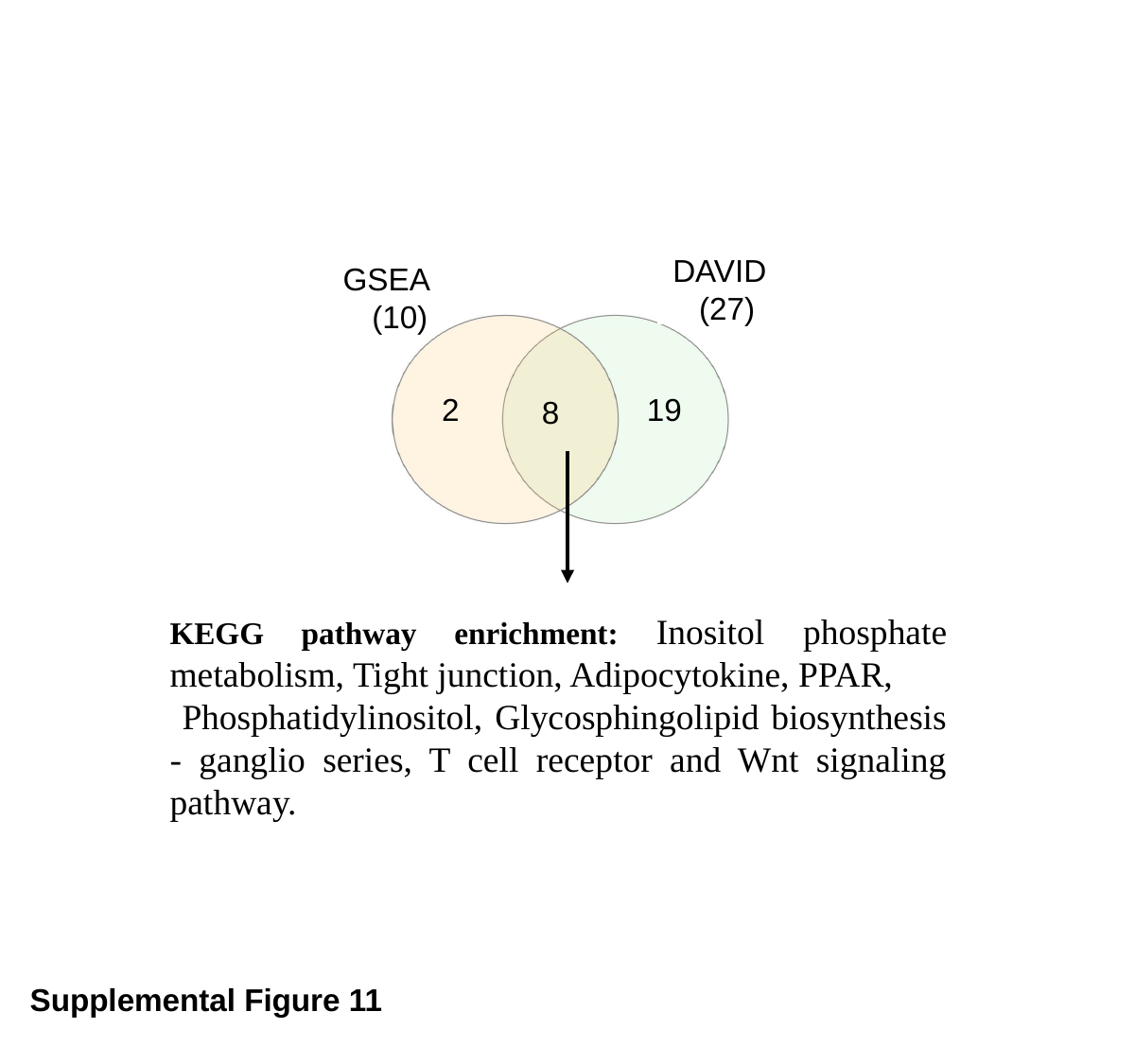

GSEA
 (10)
DAVID
 (27)
19
2
8
KEGG pathway enrichment: Inositol phosphate metabolism, Tight junction, Adipocytokine, PPAR,
 Phosphatidylinositol, Glycosphingolipid biosynthesis - ganglio series, T cell receptor and Wnt signaling pathway.
Supplemental Figure 11
