# Supplementary material for: Maternal obesity alters the placental transcriptome in a fetal sex-dependent manner
Source: Front Cell Dev Biol. 2023 Jun 15;11:1178533. doi: 10.3389/fcell.2023.1178533 (PMC10309565; doi:10.3389/fcell.2023.1178533)
Supplement: Supplementary file 30 [file Table10.DOCX]

**Supplemental Table 10: KEGG pathway enrichment analysis by GSEA. List of upregulated KEGG pathways in female placentas of obese dams compared to female placentas of the control group.**

| **Pathway name** | **No of the Genes in the overlap** | **P-value** | **FDRq-value** |
| --- | --- | --- | --- |
| MAPK signaling pathway | 16 | 5.38 e-8 | 1 e-5 |
| Toll-like receptor signaling pathway | 7 | 1.36 e-4 | 1.26 e-2 |
| Homologous recombination | 4 | 2.36 e-4 | 1.46 e-2 |
| Purine metabolism | 8 | 3.9 e-4 | 1.81 e-2 |
| Neurotrophin signaling pathway | 7 | 4.96 e-4 | 1.85 e-2 |
| GnRH signaling pathway | 6 | 8.79 e-4 | 2.72 e-2 |
| T cell receptor signaling pathway | 6 | 1.24 e-3 | 3.31 e-2 |
| Biosynthesis of unsaturated fatty acids | 3 | 1.72 e-3 | 4 e-2 |
| Nicotinate and nicotinamide metabolism | 3 | 2.22 e-3 | 4.05 e-2 |
| Small cell lung cancer | 5 | 2.34 e-3 | 4.05 e-2 |

KEGG, Kyoto Encyclopedia of Genes and Genomes; GSEA, gene set enrichment analysis; FDRq, adjusted q-value.
